# A dedicated cytoplasmic container collects extrachromosomal DNA away from the mammalian nucleus

**DOI:** 10.1091/mbc.E23-04-0118

**Published:** 2023-09-21

**Authors:** Laura Schenkel, Xuan Wang, Nhung Le, Michael Burger, Ruth Kroschewski

**Affiliations:** a Institute of Biochemistry, Eidgenössische Technische Hochschule Zürich, Otto-Stern-Weg 3, 8093 Zürich, Switzerland; b Molecular Life Science PhD Program, Life Science Zurich Graduate School, 8057 Zurich, Switzerland; National Institutes of Health, NCI

## Abstract

Expression from transfected plasmid DNA is generally transient, but it is unclear what process terminates it. We show that DNA entering mammalian cells is rapidly surrounded by a double membrane in the cytoplasm, in some cases after leaving the nucleus. This cytoplasmic container, termed exclusome, frequently also contains extrachromosomal telomeric DNA, and is maintained by the cell over several division cycles. The exclusome envelope contains endoplasmic reticulum proteins and the inner-nuclear membrane proteins Lap2β and Emerin, but differs from the nuclear envelope by its fenestrations and the absence of the Lamin B Receptor and nuclear pore complexes. Reduction of exclusome frequency upon overexpressing Emerin’s LEM-domain suggests a role for Emerin in plasmid DNA compartmentalization. Thus, cells distinguish extrachromosomal DNA and chromosomes and wrap them into similar yet distinct envelopes keeping the former in the exclusome but the latter in the nucleus, where transcription occurs.

## INTRODUCTION

In all eukaryotes the genome is enclosed in the nucleus, which compartmentalizes the chromosomes away from the cytoplasm (Güttinger *et al.*, 2009). The separation between nucleoplasm and cytoplasm is ensured by a flat-double membrane continuous with the endoplasmic reticulum (ER). Exchange between the nucleoplasm and the cytoplasm occurs mainly through the nuclear pore complexes (NPCs), which are embedded in this double membrane. This NPC-containing double membrane is further specialized by the presence of inner-nuclear membrane proteins to constitute the nuclear envelope. In many species, the nuclear envelope breaks down during mitosis and reassembles around the chromosomes upon mitotic exit establishing nuclei, where the contained DNA is replicated and transcribed. However, it is not known whether nuclear envelope assembly is restricted to the surface of chromosomes at the end of mitosis or whether it can wrap around any DNA.

How mammalian cells assemble the nuclear envelope at the end of mitosis has been intensively studied. When the separated chromosomes are pulled to opposite spindle poles towards the end of anaphase, tubular ER membranes approach each segregating chromosomal mass establishing the two new nuclear envelopes (Anderson and Hetzer, 2007; Anderson and Hetzer, 2008). Barrier-to-autointegration factor (BAF, BANF), which binds DNA in a sequence unspecific manner, accumulates at the surface of the mitotic chromosomes (Zheng *et al.*, 2000; Samwer *et al.*, 2017) and facilitates their enwrapping by assembling a nuclear envelope. It does so through binding and recruiting Lap2, Emerin, MAN1 (LEM)-domain proteins that are embedded in the ER-membrane at that stage (Haraguchi *et al.*, 2000; Haraguchi *et al.*, 2001; Lee *et al.*, 2001; Kobayashi *et al.*, 2015). NPC assembly occurs after membrane patches established contact with the chromosomes and contribute to nuclear-envelope sealing (Otsuka *et al.*, 2018; Kutay *et al.*, 2021). If these events indistinctively take place and wrap up any type of DNA or whether they are exclusive to chromosomes is unknown.

Other situations where DNA becomes enwrapped by a membrane at the end of mitosis include lagging chromosomes that remain separated from the main chromosome mass at the end of anaphase. At least initially, all characteristic hallmark proteins and protein complexes of the nuclear envelope are present in the envelope of these micronuclei, including Lamin B1 and NPCs (Hatch *et al.*, 2013; Liu *et al.*, 2018). In such compartments initially even transcription and replication take place (Hatch *et al.*, 2013). However, micronuclei degenerate over time. The enclosed DNA becomes fragmented, the envelope loses its NPCs, Lamin B1 and ultimately the DNA fragments reintegrate into chromosomes during one of the following mitoses (Crasta *et al.*, 2012; Hatch *et al.*, 2013; Zhang *et al.*, 2015). However, why the micronuclei degenerate while the nucleus stays intact is unclear. Remarkably, while lagging chromosomes or chromosome fragments are frequent, the vast majority of them reintegrate into the future nucleus during mitosis and only very few of them turn into a micronucleus (Karg *et al.*, 2015; Orr *et al.*, 2021). Together, these data indicate that separate nuclei and nucleus-like structures can form in the same cell, although in many instances the smaller structures are unstable. However, whether extrachromosomal DNA can also mediate the formation of a nucleus-like structure and thus nucleus-like envelope around them is not clear.

Two main types of extrachromosomal DNA can be found in nearly all cell types. Endogenous extrachromosomal DNA encompasses circular DNA and linear DNA fragments excised from chromosomes (Takeda *et al.*, 2001; Hoffelder *et al.*, 2004; Møller *et al.*, 2018; Paulsen *et al.*, 2018; Noer *et al.*, 2022). In contrast, extrachromosomal DNA of exogenous origin has been introduced into cells from the environment during viral or bacterial infections or during DNA transfection (Hotopp *et al.*, 2007; Watson *et al.*, 2015). To investigate whether cells assemble a nuclear envelope around extrachromosomal DNA, we have investigated the fate of plasmid DNA upon its transfection into mammalian cells. Our results provide new mechanistic insights into how cells might rapidly restrict the expression of transfected DNA.

## RESULTS

### Cells sort transfected plasmid DNA to the cytoplasm

**FIGURE 1:**
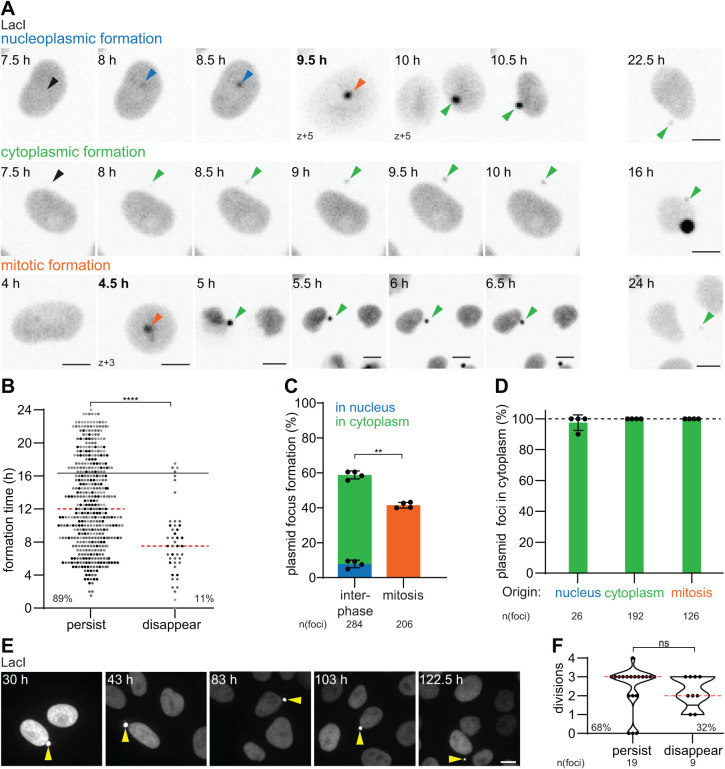
Cytoplasmic plasmid foci have three origins and are maintained in the cytoplasm long-term. (A) Time-lapse images of focus formations in HeLa-LacI cells at imaging start polymer-based transfected with pLacO. Right images: last time point a given focus was detectable. Scale bar, 10 µm. Time, after addition of DNA-Transfection reagent mix; bold time, mitosis. Arrowheads: nucleoplasmic focus, blue; cytoplasmic, green; mitosis, orange; future focus formation, black. Second cytoplasmic focus formed during mitosis in nonpresented time points, green arrow. Single z-slices. (B) Timing of individual focus formation events (circle) after addition of pLacO-Transfection reagent mix; persisting (persist) and disappearing (disappear) foci. Four experiments (exp.) pooled (exp. one, black, exp. two, dark grey, exp. three, mid grey; *n*[foci]: 490; median, red). Last 25% of all appearances, above black line. Percentage relative to all foci formed. This nonnormal data was tested with a Mann-Whitney test. **** = *p* value < 0.0001. (C) Focus formations in interphase or mitotic cells relative to all focus formations. One exp., circle; mean & SD. This normal data was tested with a paired *t* test, ** = *p* value 0.0018. (D) Foci that are in the cytoplasm at imaging end depending on their origin. Last 25% formations (in B) excluded. Color code as in (A). *n*(foci): 344; 100% reference, dashed line. (E and F) Imaging started 30 h after pLacO polymer-based transfection. One exp., *n*(cells): 28. (E) Time-lapse images of a pLacO transfected cell with one persisting focus (yellow arrowhead). Images, maximum intensity (max.) projected; scale bar, 10 µm. (F) Maximal number of divisions a focus was detectable. One focus, circle. Persisting (persist) until imaging end or disappearing before (disappear); percentage relative to all foci; median, red. This nonnormal data was tested with a Mann-Whitney test; ns = nonsignificant.

To visualize transfected plasmid DNA, we used the LacO/LacI-system, where a plasmid (called henceforth pLacO) containing 256 repeats of the Lactose operon (LacO) is introduced into HeLa cells (called henceforth HeLa-LacI) stably expressing the Lac Inhibitor protein (LacI) fused to either GFP or mCherry. Fluorescent LacI foci in the cytoplasm were detected in cells transfected with plasmid DNA either by polymer-based transfection or electroporation (two methods to introduce plasmid) and not in untransfected cells or cells transfected with a plasmid lacking a LacO array (Supplemental Figure 1; and Wang *et al.*, 2016). These results confirm that the LacI foci report plasmid localization in cells after transfection. Thus, we first used this reporter system to characterize the localization and dynamics of these plasmid foci. We performed time-lapse live-cell microscopy for up to 24 h (Figure 1, A–D, Supplemental Figures 2 and 3), starting image acquisition concomitantly with the addition of the plasmid-transfection-polymer mix to the cells and under imaging conditions that preserved viability and proliferation of the cells (Supplemental Figure 2A).

We observed the formation of plasmid foci throughout the imaging period, perhaps because plasmids continuously entered the cells (Figure 1B, Supplemental Figure 2B). Most of these plasmid foci (89%) persisted throughout the entire imaging period (Figure 1B). The other plasmid foci (11%) were visible for variable durations (between 30 min and 17 h) before disappearing (Figure 1B, Supplemental Figure 2C). Consistent with our previous study (Wang *et al.*, 2016), most cells exhibited only one plasmid focus (63% cumulative over the imaging period; Supplemental Figure 2D). Next, we analyzed the history of cells that had one focus at the end of imaging (211 cells in total, 67% of all focus cells). Interestingly, we found that 76% of these one-focus cells resulted from cells that only formed a single plasmid focus. In contrast, partitioning of foci into daughter cells during a mitosis of a cell with multi-foci cell or disappearance of foci (21% and 3%, respectively) contributed less to the cells with one plasmid focus at the end of imaging. Notably, we did not observe any plasmid foci fusion events under our imaging conditions neither in this analysis nor, when we followed individual foci in cells with multiple-plasmid foci during the imaging period (Supplemental Figure 2, E and F [total 291 plasmid foci in 114 cells over up to 24 h]). We cannot exclude that fusion events become detectable at shorter imaging intervals. These data show that transfected cells usually form only one plasmid focus. Once formed, plasmid foci are generally stable.

Our live-cell imaging also revealed that 58% of plasmid foci formed during interphase, while the other 42% were plasmid foci formed during mitosis, away from the chromosomal mass (Figure 1, A [mitotic formation, bold time point] and C, Supplemental Figure 2I). Amongst the plasmid foci formed during interphase, 88% formed in the cytoplasm and 12% in the nucleus (Figure 1, A and C, Supplemental Figure 2G). Next, we analyzed the location of each appearing plasmid focus at the latest possible time point. Irrespective of where and when plasmid foci formed, all but one ended up in the cytoplasm (Figure 1, A [at last frame] and D, Supplemental Figure 2, H and I).

Further, we wondered how plasmid foci formed in the nucleus ended up in the cytoplasm. Focusing on plasmid foci formed in the inner nucleoplasm, we observed two different translocation modes: 13 out of 15 such plasmid foci were sorted away from the chromosomes into the cytoplasm during mitosis (Supplemental Figure 3, A [first example] and B). The other two-plasmid foci left the nucleus during interphase (Supplemental Figure 3, A [second example] and B), revealing that the cell employs at least two distinct mechanisms to exclude plasmid DNA from the nucleus.

These observations make five points. First, most of the plasmid DNA remains in the cytoplasm from the outset, probably without ever reaching the nucleus. Second, there are two modes for expelling nuclear plasmid from the nucleus: through interphase sorting (Supplemental Figure 3A [second example]) or through mitotic sorting (Figure 1A upper panel 9.5 h, and bottom panel 4.5 h, Supplemental Figures 2I and 3A [second example] and B). Third, the sorting of plasmid DNA from chromosomal DNA occurs rapidly; plasmid foci formed either during mitosis or in the nucleus during interphase relocated to the cytoplasm within 1 h after their appearance (median, Supplemental Figure 3C). Fourth, in contrast to micro­nuclei formed by lagging chromosomes or parts of them, plasmid foci formed during mitosis are predominantly formed before (88% of mitotically appearing plasmid foci) and not during or after anaphase, when micronuclei become visible (Supplemental Figure 3, D and E). Furthermore, unlike micronuclei plasmid foci formed away from the region between the separating anaphase chromosomes (Figure 1A, mitotic formation 4.5 h, Supplemental Figures 3, D and E, [Wang *et al.*, 2016; Liu *et al.*, 2018; Orr *et al.*, 2021]). Therefore, we conclude that the dynamics of transfected plasmid DNA are distinct from the mitotic separation of chromosomal fragments or lagging chromosomes from the chromosomal mass. Fifth, most plasmid foci are formed during interphase (58%) and are thus not mitotic products, unlike DNA in micronuclei. Overall, these data reveal that HeLa cells have three ways to specifically sort plasmid DNA away from the nucleus and collect it in cytoplasmic foci, where it persists.

### Cytoplasmic plasmid foci remain separated from chromosomes over extended periods of time

Next, we moved the period of live cell imaging between 30 h to up to 122.5 h after addition of the DNA-transfection reagent mix and assessed if the separation between chromosomes and plasmid DNA is maintained long time after transfection (Figure 1, E and F). About two-third of the cytoplasmic plasmid foci were maintained and stayed separated from chromosomes during this period, frequently being propagated over three cell divisions in these movies. The fluorescence of about one-third of the tracked foci however decayed over more than 10 h and finally disappeared (Figure 1F, Supplemental Figure 4). Because the fluorescence decay occurred only at some plasmid foci within a whole field of view, it was not due to bleaching, but suggests that the DNA was degraded (Supplemental Figure 4). Cytoplasmic plasmid foci remained in the cytoplasm during the imaging period and we never observed that they entered the nucleus (Figure 1E, Supplemental Figure 4), unlike the DNA of micronuclei (Crasta *et al.*, 2012; Zhang *et al.*, 2015). Thus, even up to 122.5 h after transfection, plasmid foci behaved similarly to early periods after polymer-based transfection. Thus, the separation between chromosomal DNA and plasmid DNA is persistent over several divisions, consistent with our previous report (Wang *et al.*, 2016).

### Cells harboring a single cytoplasmic plasmid focus are predominant under diverse conditions

We next studied whether these observations were time- and plasmid-type-dependent and occurred in other cell types. We have previously shown that most pLacO polymer-based transfected MDCK cells (nontransformed canine-kidney cells) predominantly had one cytoplasmic focus per cell, 24 h after transfection. Here we assessed how MDCK-LacI and HeLa-LacI cells handled plasmid DNA at different times after electroporation and polymer-based transfection (Supplemental Figure 5, A–C). Two results were critical here. First, in both cell lines and employing both transfection methods, most cells with plasmid foci (grouped into classes of one and various multi-foci cells) had a single plasmid focus, regardless of the time point after transfection (3 h – 72 h; Supplemental Figures 2D and 5, B and C). Further, the analysis of the electroporation experiments shows that at 3 h most cells had more than one focus (pool of all different classes of multi-foci cells, 61%) and 39% of the cells were one-focus cells. This ratio changed over time. Notably, between 24 h and 72 h after transfection, the fraction of multi-foci cells decreased strongly, while that of the one-focus cells increased (one-focus cells: 50% at 24 h; 82% at 72 h; Supplemental Figure 5B). As this occurred in the absence of further plasmid uptake – in contrast to polymer-based transfection – the data suggests that either multi-foci cells died, plasmid-foci fused, or a single-cytoplasmic focus is selectively maintained.

We next tested whether the LacO repeat sequence had any effect on the cytoplasmic localization of transfected plasmids. For this, we polymer-based transfected plasmids with and without LacO repeats and with or without coding sequences into HeLa cells. Subsequently, we used *fluorescence in situ hybridization* (FISH) to visualize these plasmids (Supplemental Figure 5, D–F). For all tested plasmids, most focus-cells had a single cytoplasmic focus 24 h after polymer-based transfection, similar to our experiments where we visualized pLacO with LacI fluorescence (Supplemental Figure 5, E and F). These results show that plasmid DNA is preferentially maintained in a single plasmid focus in the cytoplasm of mammalian cells, regardless of the cell line, plasmid type, or transfection method.

### Plasmid DNA localizes to the cytoplasm in an ER-enwrapped compartment

**FIGURE 2:**
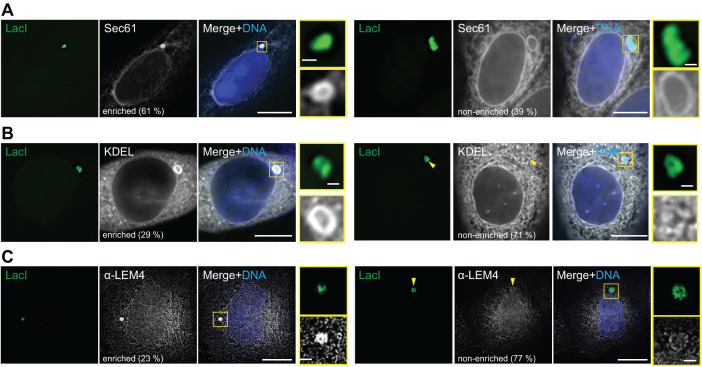
ER enwraps cytoplasmic plasmid DNA. (A–C) Representative images of the localization of ER reporters in HeLa-LacI cells 24 h after polymer-based transfection of pLacO. The frequency of two localization patterns (enriched and nonenriched, relative to the intensity of the surrounding ER). DNA, blue (Hoechst stain). Single z-slice images, deconvolved. Insets: focus; scale bars: in big images: 10 µm, in insets: 1 µm. (A) Transient expression of Sec61-mCherry. Pooled data of four exp., total *n*(cells): 76. *n*(foci): 79. Percentage relative to all foci analyzed. (B) Transient expression of GFP-KDEL. Arrowhead, position of focus; three exp.; total *n*(cells): 80; *n*(foci): 96. Percentage relative to all foci analyzed. (C) Anti-LEM4 immunostaining 24 h after polymer-based transfection of pLacO. Two exp. *n*(cells): 84; *n*(foci): 84. Percentage relative to all foci analyzed.

The cytoplasmic plasmid foci are ideal to assess if and which membrane is associated with them. As the nuclear envelope originates from the ER, and ER is abundant in the cytoplasm, we quantitatively characterized the association of plasmid DNA with ER markers. 24 h after polymer-based transfection of pLacO into HeLa-LacI cells expressing either the ER transmembrane reporter Sec61-mCherry (Figure 2A) or the ER-lumen reporter eGFP-KDEL (Figure 2B), all cytoplasmic plasmid foci colocalized with both ER reporters. Immunofluorescence detection of the ER-resident, LEM-domain protein LEM4 (ANKLE2) confirmed the presence of ER-membrane at all cytoplasmic plasmid foci 24 h after polymer-based transfection (Figure 2C) or after electroporation (Supplemental Figure 6A). The intensities of the ER-reporters KDEL and LEM4 were qualitatively similar at the plasmid focus compared with the overall ER in 71 to 91% of the cases (category referred to as “nonenriched”, Figure 2, B and C, Supplemental Figure 6A). However, the intensity of the ER-transmembrane marker Sec61 was visually enriched at 61% of the plasmid focus compared with the overall ER (Figure 2A, Supplemental Figure 6A). Thus, ER membrane and lumenal-ER proteins were minimally always present at the cytoplasmic plasmid focus.

### A distinct double membrane enwraps cytoplasmic plasmid DNA

**FIGURE 3:**
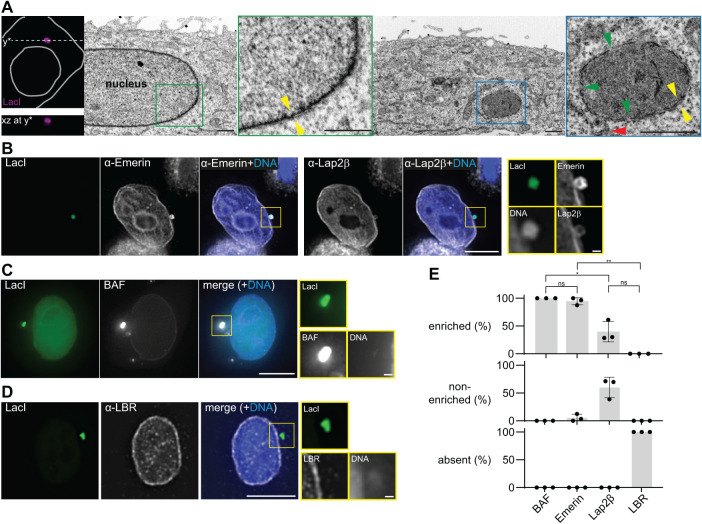
A distinct double membrane enwraps cytoplasmic plasmid DNA. (A) CLEM of an interphase cell containing one pLacO focus. Left images: confocal images; top: single z-slice xy-image superimposed with a grey cell outline and the y*-cut line; bottom: xz-view of the upper cell along the dashed y* line. The first overview EM image depicts a part of the nucleus of the cell shown in the confocal images. The second overview EM image corresponds to the same cell imaged at y*. Insets: focus, blue square; part of the interphase nucleus, green square; double-layered envelope, yellow arrowhead pair; membrane connecting proximal ER and the focus, red arrowhead; gaps in the focus envelope, green arrowheads; scale bars 1 µm. (B–D) Representative images of the localization of indicated reporters and foci in HeLa-LacI. Images: single z-slice, deconvolved; insets: foci; scale bars: in big images 10 µm; in insets: 1 µm; DNA, blue (Hoechst stain). (E) Quantification of relative localization patterns (absent, non-enriched, enriched relative to the (NE) of indicated reporters 24 h after polymer-based transfection of pLacO. Three exp. (circles); mean and SD, each with total numbers: *n*(Lap2β, foci): 52; *n*(Emerin, foci): 60; *n*(LBR, foci): 63; *n*(LBR, cells): 54; *n*(BAF, foci): 23; *n*(BAF, cells): 23. This data was tested with a Welch’s *t* test (normality could not be tested due to small *n*). ns = nonsignificant. * = *p* value 0.0298 (Lap2β vs. BAF). * = *p* value 0.0252 (Lap2β vs. Emerin). ** = *p* value 0.0015 (LBR vs. Emerin).

To visualize the cytoplasmic plasmid focus and characterize the organization of the ER-membrane around it at higher resolution, we used correlative light and electron microscopy (CLEM) in interphase HeLa-LacI cells 24 h after pLacO polymer-based transfection. In these images, a double membrane enclosing the cytoplasmic plasmid focus is clearly visible (Figure 3A, yellow arrowheads in the blue inset) as well as the nuclear envelope (yellow arrowheads in the green inset). The membrane surrounding the plasmid focus has fenestrations, indicating that it may be an open compartment (green arrowheads in the blue inset). Moreover, this membrane connects to a tube, which likely is ER (red arrowhead in the blue inset), consistent with the presence of ER proteins at plasmid foci. The cytoplasmic plasmid focus has a higher electron density than the interphase chromosomes in the nucleus suggesting denser packing of the plasmid DNA. Overall, aside from fenestration, this compartment’s membrane organization is highly reminiscent of the nuclear envelope wrapping chromosomal DNA.

To further investigate the similarities between the membrane enclosing the plasmid focus and the nuclear envelope, we probed for the presence of inner-nuclear membrane proteins at cytoplasmic plasmid foci 24 h after transfection with pLacO. We paid particular attention to those that could promote DNA-membrane tethering. One tether at the nuclear envelope is composed of BAF and LEM-domain containing inner-nuclear membrane proteins like Emerin or Lap2β (Zheng *et al.*, 2000; Lee *et al.*, 2001; Ibrahim *et al.*, 2011). Remarkably, cytoplasmic plasmid foci always contained these three proteins (Figure 3, B, C and E, [Ibrahim *et al.*, 2011; Kobayashi *et al.*, 2015]). BAF was in addition always qualitatively enriched at plasmid foci compared with the nuclear envelope suggesting a high density of plasmid molecules. More remarkable is that Emerin was enriched at nearly all foci (90%, Figure 3E, Supplemental Figure 6B, [Ibrahim *et al.*, 2011; Kobayashi *et al.*, 2015; Haraguchi *et al.*, 2022]), while Lap2β was less frequently enriched (40%; Figure 3E, Supplemental Figure 6C). Thus, LEM-domain proteins and BAF might contribute to tethering plasmid DNA to ER membranes. In contrast, the transmembrane-protein Lamin B receptor (LBR), which also links the nuclear envelope to chromatin through the heterochromatin protein 1 (Ye and Worman, 1996), was visually not detectable at plasmid foci (Figure 3, D and E). Therefore, we conclude that this system tethering heterochromatin to the nuclear envelope does not contribute to plasmid-membrane tethering. Overall, the double-membrane around the cytoplasmic plasmid focus has both similarities (presence of Emerin and Lap2β) and differences (fenestrations, absence of LBR, enrichment of Emerin) to the nuclear envelope.

### The distinct double-membrane enwrapping cytoplasmic plasmids is devoid of functional NPCs

**FIGURE 4:**
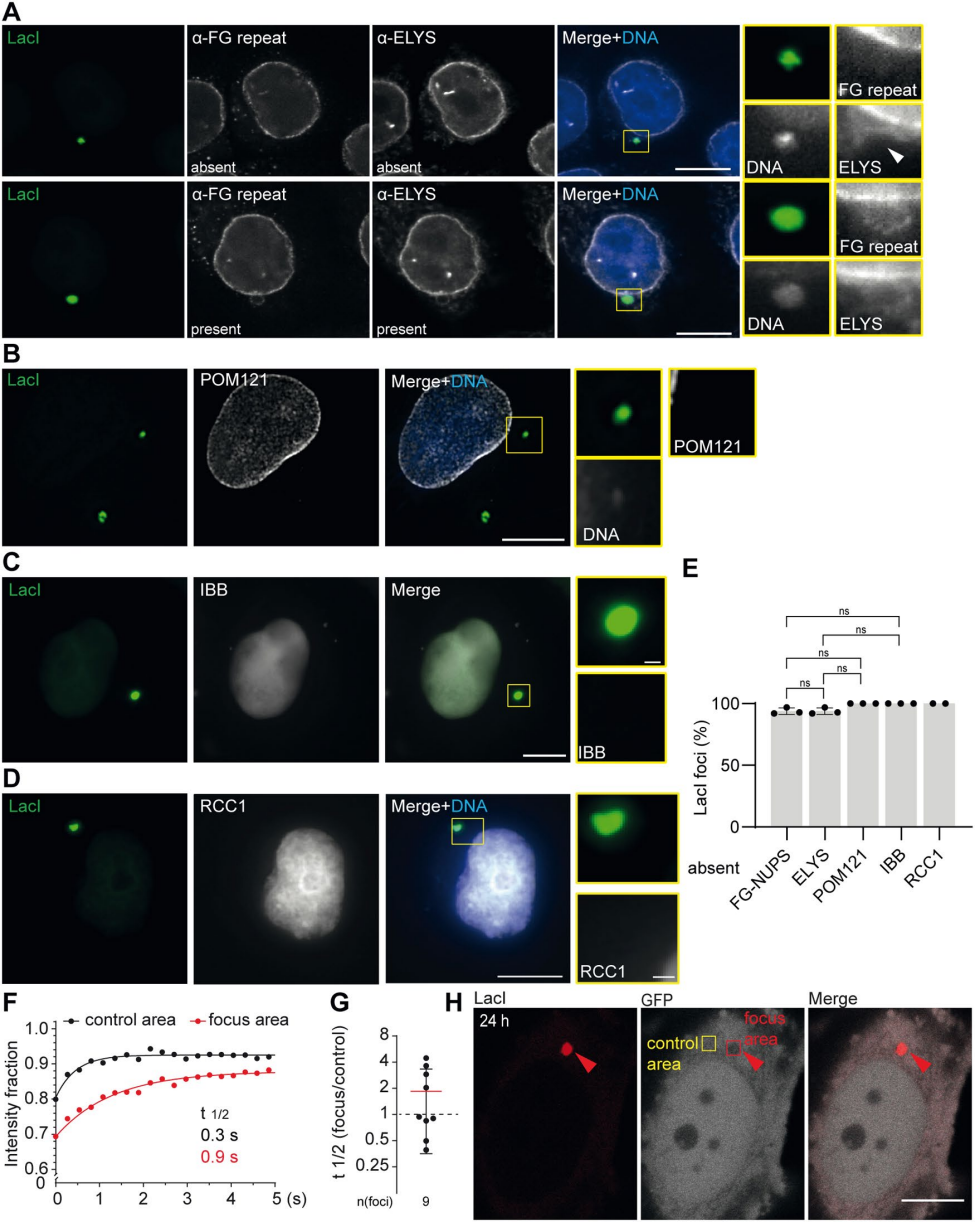
The plasmid enwrapping envelope is devoid of functional NPCs but not closed. (A–D) Representative images of HeLa-LacI cells 24 h after polymer-based transfection with pLacO. Insets: focus; scale bars: in big images: 10 µm, in insets: 1 µm; focus, arrowheads. DNA, blue (Hoechst stain). Right quantification; percentage relative to all foci; 2–3 exp., one exp., circle; mean & SD. (A) Immunostaining for ELYS and FG-repeats. Top: absence, bottom: presence example. (B–D) Images single z-slice (B) deconvolved image, POM121. (C) IBB. (D) deconvolved image, RCC1. (E) Quantification of visually inspected images in (A–D): ELYS, FG-repeats: three exp., *n*(ELYS, FG-repeats, foci): 111. POM121: three exp.; *n*(cells): 48, *n*(foci): 55. IBB: three exp.; *n*(foci): 124; *n*(cells): 87. RCC1: two exp.; *n*(foci): 55; *n*(RCC1, cells): 48. (E–G) FRAP analysis in HeLa cells transiently expressing LacI-mCherry and soluble GFP 24 h after pLacO transfection. (E) Quantification of absence of indicated markers at plasmid foci. For details on numbers see (A–D). This normal data was tested with Welch’s *t* tests. ns = non-significant. (F) Recovery of bleached GFP over time; t_1/2:_ recovery time for half of GFP intensity. (G) Quantification of (F): Ratio of t_1/2_ at focus area versus control area. Mean & SD; three exp.; one measurement, circle. *n*(foci): 9. (H) Representative images of bleaching areas. Focus area, red square; control area, yellow area. Scale bar: 10 µm.

Next, we probed for the presence of NPCs around cytoplasmic plasmid foci, 24 h after pLacO transfection and analyzed the images by visual inspection. Neither the FG-repeat containing nuclear pore proteins (FG-NUPs; anti-FG antibody) nor the Embryonic Large Molecule Derived From Yolk Sac (ELYS), a nucleoporin required for NPC assembly (Rasala *et al.*, 2006), were observed at cytoplasmic plasmid foci (94% of the cases; Figure 4A). The rare cases where these proteins were present might correspond to remnants of interphase-sorting events. Furthermore, all plasmid foci were devoid of the transmembrane protein Nuclear Envelope Pore Membrane 121 (POM121; Figure 4B). Likewise, we found no evidence for NPC-mediated, nuclear-cytoplasmic transport taking place at cytoplasmic plasmid foci. Neither the Importin β-binding Domain (IBB-GFP) nor the nucleotide exchange factor for Ran (Regulator of Chromatin Condensation 1, RCC1), which supports NPC formation and establishes a Ran-gradient across the enclosing membrane, were ever detected at cytoplasmic plasmid foci (Figure 4, C and D, [Walther *et al.*, 2003]). We conclude that the double-membrane enclosing cytoplasmic plasmid DNA is devoid of functional NPCs.

EM imaging of plasmid foci indicated that their surrounding membrane was not closed (Figure 3A). Therefore, we tested whether soluble GFP could access the cytoplasmic plasmid compartment, using Fluorescence Recovery After Photobleaching (FRAP) in HeLa-LacI cells 24 h after polymer-based transfection of pLacO. The fluorescence of LacI-GFP was bleached at the cytoplasmic plasmid focus and a reference area in the cytoplasm (Figure 4, F–H). Fluorescence recovery took place in both areas, with the recovery time (the time when half of the bleached signal is recovered, t_1/2_) being almost double for areas containing the plasmid focus (1.8 times), showing that the exchange of GFP molecules between the plasmid focus and the cytoplasm is slowed down, as expected given LacI-GFP ability to bind the LacO arrays, but otherwise very rapid. Thus, the distinct double membrane enclosing cytoplasmic plasmid DNA, while devoid of functional NPCs, still allows exchange with the cytoplasm.

### Plasmid foci forming in the cytoplasm are enwrapped by membrane within minutes

**FIGURE 5:**
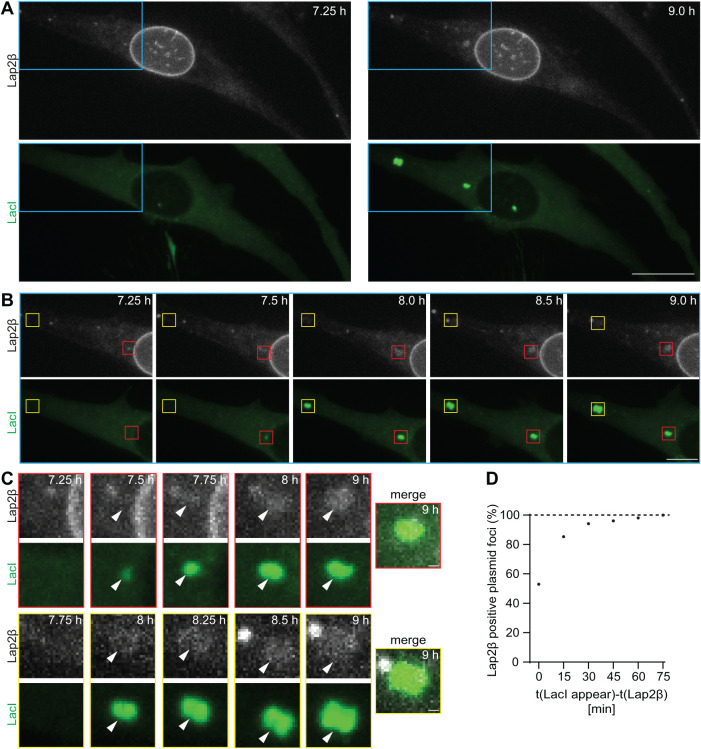
Plasmid foci forming in the cytoplasm are enwrapped by membrane within minutes. (A–C) Time-lapse images of HeLa cells stably expressing Lap2β-GFP (Lap2β) and transiently expressing LacI-mCherry (LacI) after polymer-based transfection with pLacO. Time, after pLacO transfection. (A) Overview images of the cell at 7.25 h and 9 h after transfection. The area in the blue square is enlarged in (B). Scale bar, 20 µm. (B) Enlarged part of the cell in (A). Focus forms with concomitant Lap2β association (8 h), yellow square. Focus forms and Lap2β starts to associate 15 min later (7.75 h), red square; scale bar, 10 µm. (C) Enlarged squares of (B). Focus, arrowhead; yellow squared row: Lap2β channel boosted, nonboosted images in (A and B); scale bar, 1 µm. (D) Cumulative fraction of foci associated with Lap2β in dependence on the duration of Lap2β association after focus appearance. One exp., *n*(foci): 105; *n*(cells): 49.

The nuclear membrane rapidly encloses chromosomal DNA at the end of mitosis. Therefore, we assayed the time-scale of membrane association with cytoplasmic plasmid DNA. We used HeLa cells stably expressing Lap2β-GFP and transiently expressing LacI-mCherry lacking an NLS. This ensured that nothing limited LacI binding to the plasmid, allowing early visualization of plasmid in the cytoplasm. In addition, the cells were synchronized in S phase using thymidine to ensure a higher homogeneity of the cell population. These cells were transfected with pLacO and imaged every 15 min for 25 h, starting after the addition of the polymer-based transfection-DNA mix. The appearing plasmid foci and their association with the membrane marker Lap2β-GFP was then scored (Figure 5). 51% of the plasmid foci were already associated with the membrane marker at the time of their appearance (Figure 5D). This fraction increased over time, reaching 97% of the plasmid foci after 75 min (Figure 5D). Thus, membrane association is largely concomitant with the emergence of the plasmid focus (Figure 5, C and D).

### Emerin is enriched at cytoplasmic plasmid compartments in primary human cells

**FIGURE 6:**
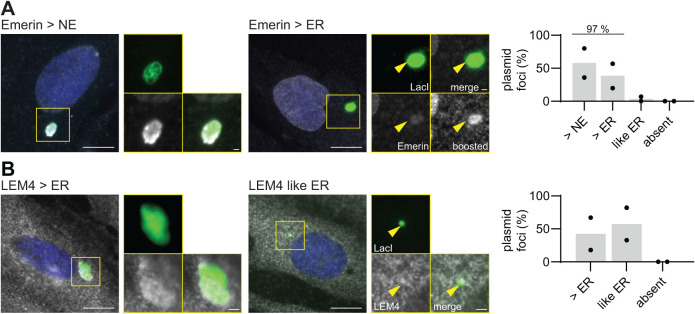
Exclusomes containing plasmid DNA exist in primary human fibroblasts. (A and B) Primary human fibroblasts 48 h after polymer-based transfection with pLacO and plasmid encoding LacI-NLS-GFP, immunostained for Emerin (A) and LEM4 (B). Single z-slice images. Insets: focus; scale bars: in big images 10 µm; in insets: 1 µm; DNA, blue (Hoechst stain). (A) Representative images of indicated classification. Plasmid focus, arrowhead. Right graph: Emerin’s intensity at the focus is greater than that of Emerin at the NE (> NE) or the ER (> ER), or like that of Emerin in the ER (like ER) in cells with one cytoplasmic plasmid focus. Two exp., One exp., circle; bar, mean; *n*(foci): 29. Emerin’s signal was boosted, boost. (B) Representative images for indicated classification of LEM4. Right graph: LEM4’s intensity at focus is greater than that of LEM4 in ER (> ER) or like that of the ER (like ER) in cells with one cytoplasmic plasmid focus. Two exp., one exp., circle; bar, mean; *n*(foci): 38.

To probe if a double membrane also enwraps cytoplasmic plasmid foci in nonimmortalized cells, we transfected primary human fibroblasts with pLacO and visualized the plasmid with transiently expressed LacI-NLS-GFP. In addition, we immunostained the cells for Emerin or LEM4 (Figure 6, A and B). We noticed that these primary cells divided significantly less frequently than HeLa cells. Therefore, we analyzed the cells 48 h after pLacO transfection. Most of these cells showed also a single plasmid focus upon transfection (Supplemental Figure 6D). Emerin was present at each plasmid focus and was even enriched at 97% of them, compared with the surrounding ER or nuclear envelope (Figure 6A). Similarly, LEM4 was always present in cells with one plasmid focus and, in 45% of these cells, even enriched compared with the surrounding ER (Figure 6B). This is qualitatively similar to HeLa cells.

Thus, both in primary human fibroblasts as well as in HeLa cells, plasmid DNA is excluded from the nucleus and localizes to the cytoplasm where it is enwrapped predominantly in one membranous organelle. We term this container the exclusome. A diagnostic hallmark of the exclusome is that Emerin and BAF are enriched as compared with the nuclear envelope and nucleus, respectively. The exclusome-envelope is further characterized by the presence of fenestrations, Lap2β and ER-membrane proteins, such as Sec61 and LEM4, whereas NPCs and LBR are absent.

### Overexpression of Emerin’s LEM-domain reduces the compartmentalization of plasmid DNA in the cytoplasm

**FIGURE 7:**
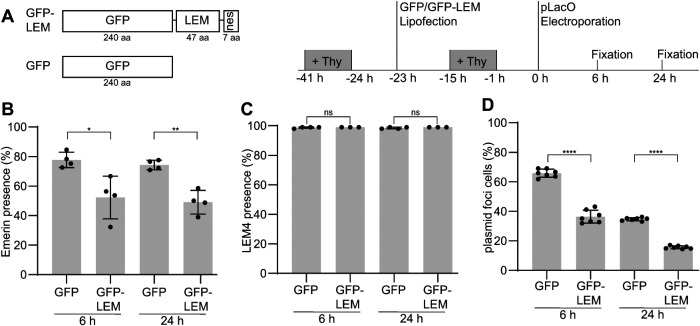
Overexpression of Emerin’s LEM-domain reduces cells with plasmid foci. (A) Scheme of fusion proteins transiently overexpressed in HeLa-LacI cells (left side) and experimental procedure (right side). GFP-LEM-nes (“GFP-LEM”), soluble GFP (“GFP”); aa: amino acid residues. + Thy: thymidine treatment; time, relative to pLacO transfection. (B) Presence of Emerin at cytoplasmic foci 6 h and 24 h after electroporation of pLacO. Four exp., one exp., circle; mean and SD; this normal data was tested with Welch’s *t* test; *: *p* = 0.0325, **: *p* = 0.044; *n*(foci): GFP 6 h: 589; GFP 24 h: 458; GFP-LEM 6 h: 474; GFP-LEM 24 h: 413. (C) Presence of LEM4 at cytoplasmic foci 6 h and 24 h after electroporation of pLacO. Three to five exp. one exp., circle; mean and SD; this normal data was tested with Welch’s *t* test; ns = nonsignificant; *n*(foci): GFP 6 h: 737; GFP 24 h: 536; GFP-LEM 6 h: 334; GFP-LEM 24 h: 280. (D) Frequency of cells containing at least one cytoplasmic focus in GFP and GFP-LEM expressing cells 6 h and 24 h after electroporation. Seven exp., one exp., circle; mean and SD; this normal data was tested with Welch’s *t* test; **** *p* < 0.0001; each with total numbers: *n*(cells): GFP 6 h: 1428; GFP 24 h: 1460; GFP-LEM 6 h: 1471; GFP-LEM 24 h: 469.

Because Emerin is enriched at ∼90% of plasmid foci and the formation of a double membrane is concomitant with plasmid-focus formation (Figures 3B, E and 5), we speculated that Emerin-dependent tethering of the plasmid DNA to the surrounding membrane may contribute exclusome formation or stabilization. As Emerin’s LEM-domain binds BAF and BAF binds DNA (Lee *et al.*, 2001), we aimed to interfere with this molecular linkage. To do so, we used a competition approach and overexpressed Emerin’s soluble LEM-domain fused to GFP and a nuclear exclusion signal (GFP-LEM). Control cells expressed soluble GFP alone (GFP; Figure 7A, left side). Subsequently, pLacO was electroporated and cells that expressed GFP-LEM or GFP were analyzed (Figure 7A, right side). Validating this competition approach, Emerin was less enriched at the nuclear envelope and more present in the ER in cells expressing GFP-LEM compared with control cells expressing GFP (Supplemental Figure 7, B and C). This suggests that GFP-LEM overexpression competed with endogenous Emerin for BAF binding and therefore DNA binding or another cause affecting the retention of Emerin in the nuclear envelope. Furthermore, Emerin associated also less frequently with cytoplasmic plasmid foci after pLacO transfection in cells overexpressing GFP-LEM compared with control (Figure 7B; Supplemental Figure 7D).

The amino acid sequences of the LEM-domains of Emerin and LEM4 are 44% similar (Supplemental Figure 7A). Because of this, the overexpressed LEM-domain of Emerin might also interfere with the function of other LEM-domain proteins. To test for this possibility and to further characterize the plasmid focus membrane, we probed for the presence of LEM4. The association of LEM4 was not affected in GFP-LEM expressing cells at either 6 h or 24 h after pLacO electroporation (Figure 7C, Supplemental Figure 7E). Moreover, LEM4 was present in all conditions at almost all cytoplasmic plasmid foci (99%, mean of three to five exp., all conditions), revealing that even Emerin-negative plasmid foci are membrane-enclosed. Furthermore, the presence of LEM4 in Emerin-negative plasmid foci indicates that LEM-domain proteins other than Emerin might not be out competed by overexpression of Emerin’s LEM-domain.

Because we could interfere with Emerin’s function, we characterized the effects of GFP-LEM overexpression on plasmid handling. In the GFP-LEM condition, fewer cells contained at least one cytoplasmic plasmid focus compared with control, both 6 h and 24 h after pLacO transfection (36% for GFP-LEM and 66% for GFP; Figure 7D). At 24 h after transfection, the number of cells with plasmid foci were halved in both conditions compared with 6 h due to cell division and asymmetric partitioning of the plasmid foci (16% for GFP-LEM and 35% for GFP). Thus, we concluded that Emerin, through its LEM-domain, supports the compartmentalization of plasmid DNA into a cytoplasmic exclusome in mammalian cells.

### Exclusomes can contain telomeric DNA

Next, we wondered whether the exclusome collected only and specifically DNA of exogenous origin or also extrachromosomal DNA excised from the chromosomes. Circular extrachromosomal DNA of telomeric origin (tDNA) is abundant in cells undergoing alternative lengthening of telomeres (ALT), like the osteosarcoma cell line U2OS (Cesare and Griffith, 2004). Remarkably, in U2OS and several other cancer cell lines, such as WI38-VA13, SaOs2*,* and KMST-6, between one and four FISH signals of extrachromosomal tDNA were detected in the cytoplasm (Tokutake *et al.*, 1998; Chen *et al.*, 2017). In addition, several groups have detected circular extrachromosomal tDNA in non-ALT cancer cells like HeLa (Tokutake *et al.*, 1998; Regev *et al.*, 1999; Wang *et al.*, 2004; Vidaček *et al.*, 2010). Therefore, we first tested whether this extrachromosomal DNA of endogenous origin is membrane-enclosed in the cytoplasm and whether this membrane shares similarities with the exclusome’s envelope.

**FIGURE 8:**
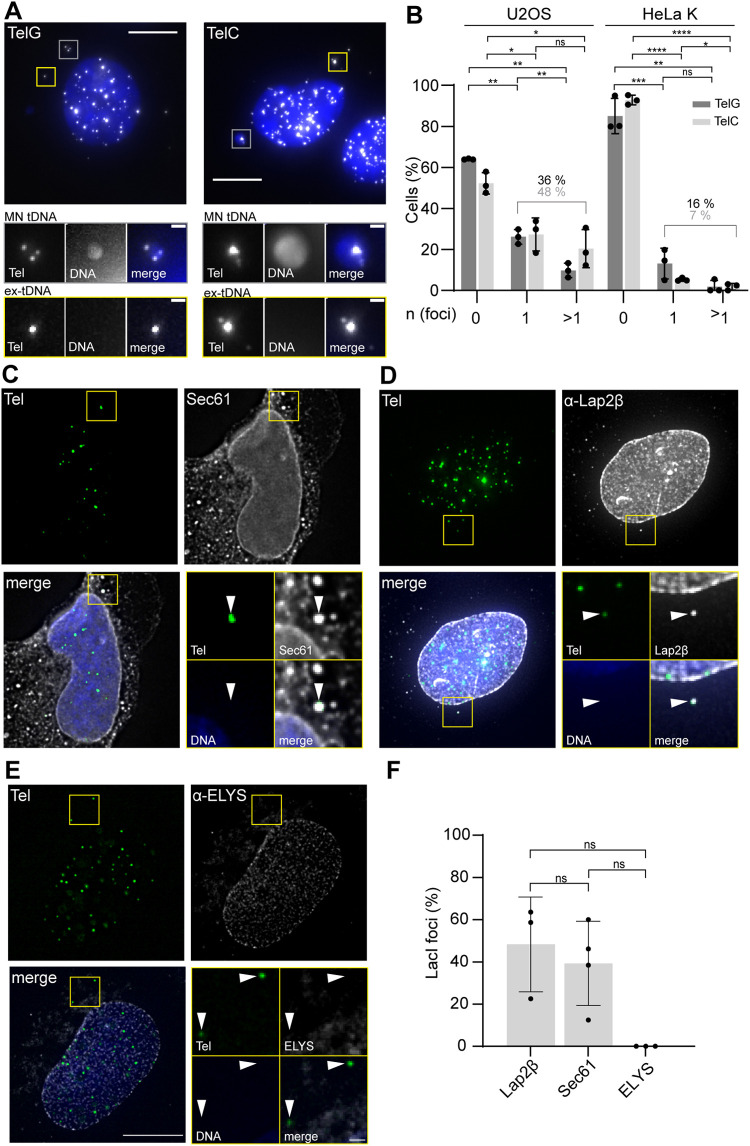
A distinct envelope enwraps also cytoplasmic extrachromosomal tDNA. (A) Representative images of U2OS cells FISH-stained with TelG and TelC probes. Images max. projected, insets: area with ex-tDNA, yellow squares; area with MN tDNA, gray squares. Scale bars: in big images: 10 µm; in insets: 1 µm. DNA, blue (Hoechst stain). (B) Frequency of HeLa K and U2OS cells with none, one (1), or more than one (>1) ex-tDNA focus relative to the total cells analyzed. Single experiment, one circle. U2OS-TelG probe: three exp.; *n*(cells): 217; HeLa-TelG probe: three exp *n*(cells): 210; HeLa-TelC probe: three exp., *n*(cells): 209. (C–E) Representative single z-slice images of FISH-IF stained U2OS cells depicting the localization of the reporter proteins (left); quantification of colocalization of respective marker at ex-tDNA focus (right, plot). Big images: max. projected deconvolved; arrowheads, ex-tDNA foci; areas of tDNA foci, insets. Scale bars: big images: 10 µm, insets: 1 µm; three exp.; DNA, blue (Hoechst stain). Signals of TelG probe, overexpressed Sec61-mCherry (C) and indicated antibodies (D and E). Percentage relative to all ex-tDNA foci analyzed. Mean & SD. Sec61, four exp.; one exp., circle; *n*(Sec61, ex-tDNA): 95; Lap2β, three exp.; one exp., circle; *n*(Lap2β, ex-tDNA): 154; ELYS, three exp.; one exp., circle, *n*(ELYS, ex-tDNA): 66. (F) Quantification of presence of indicated marker at plasmid foci. For numbers see (C–E). This normal data was tested with Welch’s *t* tests; ns = nonsignificant.

We performed FISH experiments in U2OS and HeLa cells using two different fluorescently tagged telomeric probes (TelC and TelG). In all cases, we observed numerous FISH signals in the nuclei and few in the cytoplasm (Figure 8, A and B; Supplemental Figure 8, A and B). The cytoplasmic Tel FISH signals were not caused by artifactual probe clustering because cells simultaneously hybridized with both a telomeric probe as well as a scrambled probe only showed telomeric probes signals (Supplemental Figure 8, A–C). Further, nuclear and cytoplasmic telomeric FISH signals indeed labeled DNA, as DNaseI treatment before probe hybridization abolished the signal (Supplemental Figure 8, D and E).

Micronuclei with chromosomal fragments occur frequently in cancer cells. To exclude such compartments from our analysis, we chose Hoechst fluorescence as a criterium, as the median size of circular extrachromosomal tDNA is only 5 kb (Cesare and Griffith, 2004). Based on this criterium, we distinguished two types of tDNA in the cytoplasm of HeLa or U2OS cells: tDNA in Hoechst positive foci, termed micronuclear tDNA (MN tDNA, Figure 8A [grey squares]) as they might contain chromosomal DNA fragments with telomeres; and extrachromosomal tDNA without Hoechst stain, termed ex-tDNA. There were fewer cells with cytoplasmic ex-tDNA (Figure 8A [yellow squares]) in the population of HeLa cells (7–16%) compared with U2OS cells (36–48%, Figure 8B). This is consistent with ALT cells producing more circular extrachromosomal tDNA than non-ALT ones, as expected, and with ex-tDNA foci containing circular extrachromosomal tDNAs. Remarkably, in striking similarity to transfected plasmid DNA (Wang *et al.*, 2016) both HeLa and U2OS cells mostly contained one ex-tDNA focus per cell for both probes (U2OS 25%, HeLa 5%, Figure 8B).

Thus, we analyzed next whether cytoplasmic ex-tDNA foci are wrapped in membranes. We could not directly probe for the presence of Emerin, as none of our anti-Emerin antibodies sustained the conditions used in FISH-IF experiments. However, visual analysis of the images showed that overexpressed Sec61-mCherry colocalized with 47% of ex-tDNA foci but always colocalized with MN tDNA (Figure 8, C and F, Supplemental Figure 8F) in U2OS cells. Also, Lap2β was present at 41% of ex-tDNA foci but always present at MN tDNA foci (Figure 8, D and F, Supplemental Figure 8F). ELYS was never present at ex-tDNA foci but was typically present at MN tDNA (two out of three cases; Figure 8, E and F, Supplemental Figure 8F). Thus, the colocalization frequencies for the tested ER- and inner-nuclear membrane proteins at ex-tDNAs were lower as for plasmid foci, possibly due to the reduced focus size (Figure 8, A, C–E, yellow squares) and the harsh conditions applied during FISH. Notably, NPCs were absent from ex-tDNA but not from MN tDNA (Figure 8E, Supplemental Figure 8F), consistent with the possibility that the latter are formed during mitosis and have different contents than ex-tDNA foci. Collectively, these results indicate that ex-tDNA can also be contained in exclusomes.

### Both plasmid DNA and ex-tDNA cluster in the same exclusomes

**FIGURE 9:**
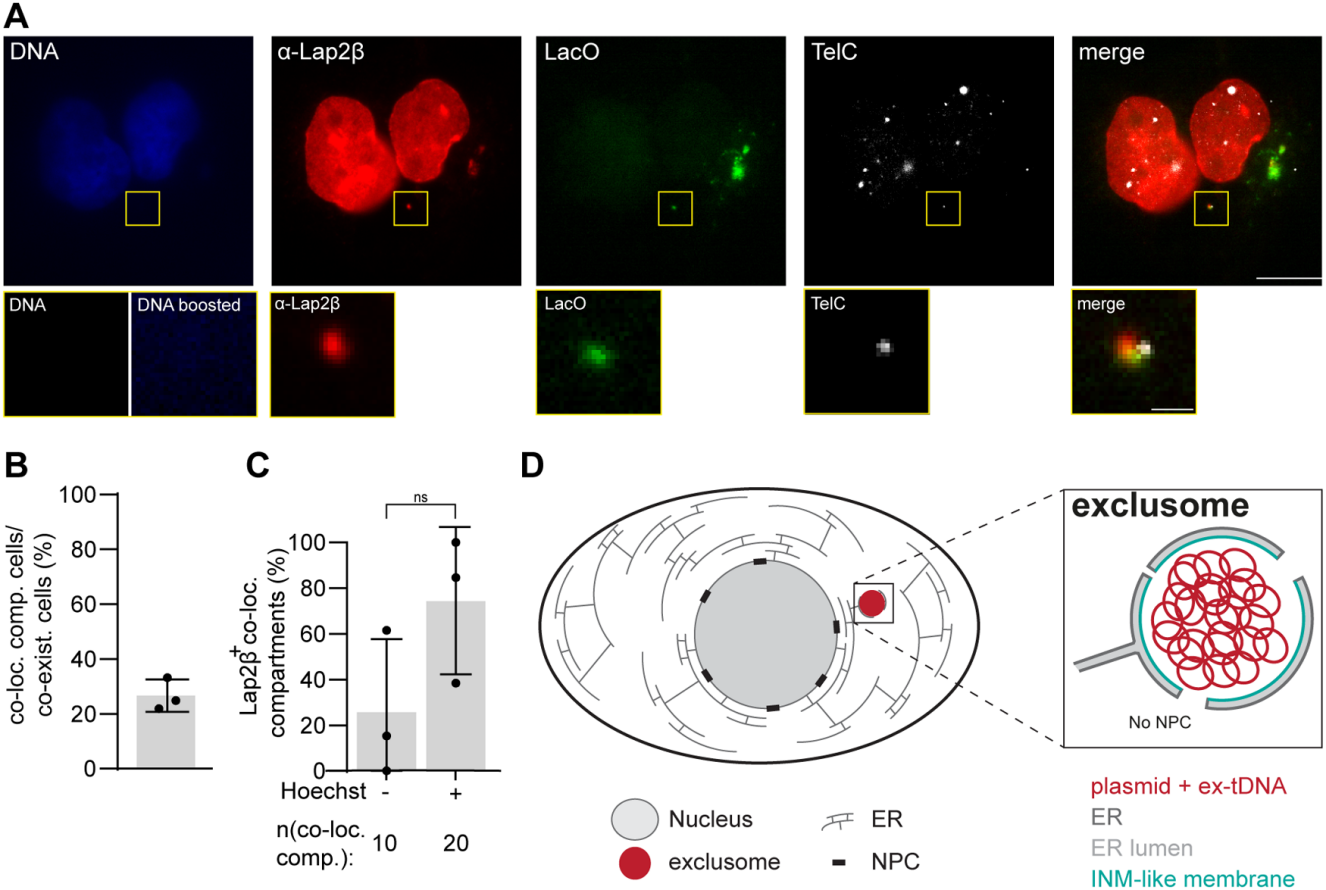
Plasmid DNA and ex-tDNA can be clustered in one exclusome. (A) Representative image of a U2OS cell transfected with pLacO, fixed, hybridized with TelC and LacO probes and immunostained against Lap2β. DNA, blue (Hoechst stain). Inset: cytoplasmic compartment with two DNA species; DNA stain inset boosted, DNA boosted. Scale bars: in big images: 10 µm, in insets: 1 µm. (B) Frequency of U2OS cells with minimally one colocalization compartment in cells, which contain both DNA species (coexistence cells) 24 h after pLacO transfection. One experiment, circle *n*(coexistence cells): 155; *n*(coexistence cells): 40. (C) Frequency of Lap2β positive cytoplasmic colocalizing compartments. 24 h after pLacO transfection. One experiment, circle *n*(Lap2β+ colocalizing compartment): 40. This normal data was tested with paired *t* test, ns = nonsignificant. (D) Model of an exclusome in an interphase cell. Overview of a cell with nucleus, ER and exclusome (inlet: the magnified exclusome with details).

Due to the observed similarities between ex-tDNA and plasmid DNA, we tested whether they colocalize within the same exclusomes. U2OS cells were fixed and immunostained for Lap2β 24 h after polymer-based transfection with pLacO and analyzed by visual inspection. In addition, the cells were hybridized with probes for both LacO and TelC. Indeed, plasmid and ex-tDNA colocalized in the cytoplasm in one Lap2β containing membrane compartment (Figure 9A). Among all cells with both types of DNA in the cytoplasm (coexistence cells), 26% had both DNA types in a single cytoplasmic compartment (Figure 9B). 75% of such compartments contained Lap2β in their envelope (Figure 9, A and C). In 74% of such Lap2β-containing compartments plasmid and tDNA foci were Hoechst positive, which could represent chromosomal fragments with telomers together with plasmid DNA (Figure 9C). However, the fact that 26% of such compartments were Hoechst negative reveals that ex-tDNA, and not fragments of chromosome ends with telomeric DNA, colocalized with plasmid DNA in one cytoplasmic membrane-bound compartment (Figure 9A). Therefore, we conclude that extrachromosomal DNAs of different origins, such as endogenous telomeric DNA and exogenous plasmid DNA, can cluster in the same exclusomes (Figure 9D).

## DISCUSSION

In animal cells, transfected DNA is expressed only transiently but the mechanisms of its silencing are not very clear (Wang *et al.*, 2016; Fus-Kujawa *et al.*, 2021). Here we investigated the fate of the transfected DNA. We show that the cell collects a large fraction of this DNA in a dedicated cytoplasmic container, which we named the exclusome. The DNA targeted to the exclusome may come either from the cytoplasm or the nucleus. Furthermore, the exclusome collects endogenous DNA excised from the chromosomes, such as tDNA. An exclusome is delimited by a sheet-like double membrane marked with ER-membrane proteins as well as at least the inner-nuclear membrane proteins Emerin and Lap2β, all characteristics shared with the nuclear envelope (LaJoie and Ullman, 2017). In contrast to the nuclear envelope, the envelope of the exclusome does not contain NPCs, LBR and remains fenestrated (Franke *et al.*, 1981; Ye and Worman, 1994; Otsuka *et al.*, 2018; Ventimiglia *et al.*, 2018).

These similarities and distinctions between the exclusome and the nuclear envelopes are interesting in two different manners. The similarities between these two structures indicate that any DNA present in the cytoplasm is initially being recognized by the same core machinery. This core machinery ensures that the DNA is being 1) clustered and 2) enwrapped in membrane such as to form each time a single entity in the cell, a single nucleus and a single exclusome, or nearly so. This minimal envelope formed in both cases suggests that the investigation of how cells enwrap exogenous DNA upon transfection might be useful also for understanding the early events of nuclear envelope formation at the end of mitosis (Haraguchi *et al.*, 2000; Haraguchi *et al.*, 2001; Anderson and Hetzer, 2008; Haraguchi *et al.*, 2008; Lu *et al.*, 2011). Interestingly, we note, that exclusome assembly is not itself a cell-cycle dependent process and can take place in interphase as well as throughout mitosis. Thus, DNA clustering and formation of an initial minimal DNA envelope are not dependent on cell-cycle regulation. Further, it is tempting to speculate that the physiological role of exclusome assembly does not only serve to collect and confine exogenous DNA away from the nucleus, where transcription occurs, in general but might also help to control potential pathogens at early stages of infection. In turn, the fact that many DNA viruses, such as mimivirus and vaccinia, proliferate in ER-associated replication factories may represent how some viruses have learned to hijack this defense to their own advantage (Mutsafi *et al.*, 2013; Greseth and Traktman, 2022).

However, the most striking and probably functionally most relevant elements of this comparison are the differences that distinguish the exclusome from the nuclear envelope, namely the absence of NPCs, LBR, and a controlled exclusome-cytoplasmic exchange. Thus, the envelope of the exclusome might be comparable to a nuclear envelope that would have stopped its maturation before NPC assembly (Haraguchi *et al.*, 2000; Otsuka *et al.*, 2018; Otsuka *et al.*, 2023). Interestingly, the protein composition of the exclusome recapitulates that of the “core region” of the nuclear envelope during its reassembly at the end of mitosis, whereas the excluded proteins correspond to those specific of the “noncore region” (Haraguchi *et al.*, 2000). Strikingly, the capacity of the nucleus to assemble the complex machineries involved in gene expression depends on the nucleocytoplasmic exchange (Buchwalter *et al.*, 2019). Thus, the collection of exogenous DNA in the exclusome might serve in the first place in silencing it. In support of this idea, while expression of the transfected DNA remains very transient, the exclusome persists, indicating that its content is not expressed.

Together, our findings open the question of how cells distinguish between chromosomes and exogenous DNA to assemble a full and functional envelope around the first ones and enwrap the latter only with a minimal, fenestrated and passive one. On one hand, LEM-domain proteins seem to play an interesting role in this process. Thus, understanding the relative roles of the different LEM-domain proteins of mammals in exclusome biology will require more attention. Our data suggest that Emerin may play a fundamental role, more than Lap2β. However, other LEM proteins such as Lap2α and MAN1 would also deserve attention. Furthermore, given the interaction of these proteins with BAF, given the role of BAF in DNA clustering, envelope assembly, and in cellular responses to viral infection (Zheng *et al.*, 2000; Kobayashi *et al.*, 2015; Oh Hyung *et al.*, 2015; Wiebe Matthew and Jamin, 2016), and given the abundance of BAF in the exclusome, BAF may also play a fundamental role at the exclusome. Therefore, it is attractive to postulate that many of the exclusome components are acting together in a pathway dedicated to the recognition, clustering, and envelopment of exogenous DNA. The proposed role of Emerin in the export of cytomegalovirus particles out of the nucleus support our idea (Milbradt *et al.*, 2014). On the other hand, we noticed the absence of RCC1 and ELYS from exclusomes. Given the crucial roles of these proteins in NPC assembly and the establishment of active exchange between the nucleoplasm and cytoplasm, their absence alone would explain why the exclusome-envelope remains poreless and fenestrated and without LBR (Walther *et al.*, 2003; Gómez-Saldivar *et al.*, 2016; Mimura *et al.*, 2016). Further studies will be required for determining why exogenous DNA, including the DNA that has been in the nucleus, fails to recruit these factors. It is tempting to speculate that the chromatinization level in somatic cells will play an important role, as it was shown for mouse germ line pronuclei (Inoue and Zhang, 2014). However, we would like to speculate that the chromatin status, which ensures the recruitment of factors such as RCC1 and ELYS onto chromosomes and prevents chromosomes from being expelled to the exclusome, might require specific signals, possibly coming from the centromere. Indeed, human artificial chromosomes (HAC) relocalize to a cytoplasmic “nanonucleus” upon inactivation of their engineered centromere (Nakano *et al.*, 2008). These nanonuclei have not been further characterized. A role for the centromere would be reminiscent to the situation described in budding yeast, where the centromere was found to function in distinguishing chromosomal from extrachromosomal DNA (Kruitwagen *et al.*, 2018).

Of note, and supporting our speculations above, our data clearly indicate that the exclusome is not a micronucleus. Micronuclei only form at the end of mitosis. Exclusomes however form mainly in interphase. If they form during mitosis, then predominantly before anaphase and not in the area between the separated chromosomal masses. In addition, exogenous DNA that reached the nucleus was excluded from it either in early mitosis, or during interphase, as also observed for chromosome-derived large circular DNAs encoding c-myc (Shimizu *et al.*, 1998). Unlike the content of micronuclei that can reintegrate into the nucleus (Zhang *et al.*, 2015), we did not observe this for exclusome DNA. Furthermore, Emerin was strongly enriched in the exclusomes envelope relative to the nuclear envelope, which is not observed in micronuclei (Liu *et al.*, 2018). An important distinction between exogenous DNA or extrachromosomal DNA of endogenous origin found in the exclusome and the large chromosome fragments or entire chromosomes found in micronuclei is that the latter ones are still or where recently connected to a centromere and have the typical chromatin organization of mitotic chromosomes. This might be paramount to their ability to support the assembly of a functional envelope around them (Hatch *et al.*, 2013). Thus, our data indicate that mammalian cells, like yeast, can distinguish self or chromosomal from nonself or extrachromosomal DNA. It will be interesting to dissect how they do so and to determine how the subsequent clustering of these two distinct materials away from each other is brought about.

Finally, if the function of the exclusome is to extract, confine, and silence exogenous DNA in mammalian cells, it should come as a surprise that its DNA is not subsequently degraded or eliminated. We anticipate that in the context of a multicellular organism in which cells had formed an exclusome, it is the role of the organismal immune system to take care of such an elimination. Thus, in this context the exclusome might serve a signaling hub. By recruiting cyclic guanosine monophosphate-adenosine monophosphate synthase (cGAS), which provokes alarming type I interferon production, a persistent exclusome would ensure that the affected cell becomes eradicated after a while (Ablasser *et al.*, 2013; Wan *et al.*, 2020). As cGAS was found at transfected cytoplasmic plasmid DNA, the persistence of the exclusome might serve to make sure that it is indeed the case (Guey *et al.*, 2020). Additionally, an exclusome might alter the cellular reaction to other incoming DNAs and could explain why transfection or virus infection are less efficient subsequently to a first transfection (Grandjean *et al.*, 2011; Langereis *et al.*, 2015). In such a scenario, we suggest that the exclusome might act as a memory deposit for both organismal and cell-autonomous immunity towards DNA.

In summary, our study starts to unveil the existence of sophisticated, cell-autonomous mechanisms of native immunity against at least exogenous DNA. This machinery might have been inherited from the distant past, a time at which our ancestors were still unicellular organisms. It might therefore be closely related to what is found in fungi and other nonmetazoan eukaryotes. Future studies will determine how it works, has evolved and is now coordinated with the organismal systems of immunity that multicellularity has allowed to emerge.

## MATERIALS AND METHODS

### Mammalian cell lines

All cell lines listed in the following were cultured at 37°C with 5% CO_2_ in a humidified incubator in the indicated media. Cell lines were tested for mycoplasma every 6 mo.

**Table:** 

Name	Stable transgene	Transient transgene	internal Lab ID	Culture medium	Used in
HeLa Kyoto (HeLa K)	–	–	MMC278	DMEM +10% FCS +P/S	Figure 8, A and B, Supplemental Figure 8, A and C
HeLa LacI-NLS-mCherry	LacI-NLS-mCherry	–	MMC114	DMEM +10% FCS +P/S +Blasticidine	Figures 2, B and C, 3, A and C, 4, B–D, 7, Supplemental Figures 1 and7
HeLa LacI-NLS-GFP	LacI-NLS-GFP	–	MMC105	DMEM +10% FCS +P/S +Blasticidine	Figure 1, E and F, 2A, 3B and D, 4A, Supplemental Figures 1, 4, and 5, C, E, and F
Hela K	–	LacI-mCherry and eGFP	–	DMEM +10% FCS +P/S +Blasticidine	Figure 4, E–G
HeLa LacI-NLS-GFP mock electroporated = Control HeLa	LacI-NLS-GFP	–	MMC248	DMEM +10% FCS +P/S +Blasticidine	Figure 1, Supplemental Figures 1, 2, and 3
HeLa K Lap2β-GFP	aa 244–453 of Lap2β-GFP	LacI-mCherry	MMC84	DMEM +10% FCS +P/S	Figure 5
MDCK LacI	LacI-NLS-EGFP	–	MMC100	GlutaMax 10% FCS +P/S	Supplemental Figure 5, A–C
U2OS	–	–	MMC95	DMEM +10% FCS +P/S	Figures 8 and 9, Supplemental Figure 8
Human primary foreskin fibroblasts	–	LacI-NLS-GFP	MMC281	DMEM +10% FCS +P/S	Figure 6, Supplemental Figure 6C

 

#### HeLa.

HeLa Kyoto (HeLa K) human-cervical cancer cells were a kind gift from P. Meraldi (ETHZ, Switzerland) and originated from S. Narumiya, (Kyoto University, Japan).

HeLa K cells stably expressing a mutant form of LacI that cannot tetramerize in the fusion proteins LacI-NLS-mCherry and LacI-NLS-EGFP were described in (Wang *et al.*, 2016). Both LacI-NLS-XFP cell lines, as well as HeLa transiently expressing LacI-mCherry are referred to “HeLa-LacI” throughout the result and legend text.

HeLa K cells stably expressing LacI-NLS-EGFP which were in addition mock electroporated, is termed ”Control HeLa” in Supplemental Figure 1 but otherwise “HeLa-LacI” to facilitate readability.

Cell lines HeLa-LacI and HeLa K were authenticated by analysis of PCR results over characteristic highly polymorphic short tandem repeat loci (Microsynth; Balgach, Switzerland).

HeLa K cells stably expressing aa 244– 453 of Lap2β-GFP were kindly provided by U. Kutay and originated from (Mühlhäusser and Kutay, 2007). Expressed aa 244– 453 of Lap2β-GFP is termed “Lap2β.”

#### MDCK (Madin Darby canine kidney cells).

MDCK II cells stably expressing LacI-NLS-EGFP (MDCK-LacI), were described in (Wang *et al.*, 2016).

#### U2OS (human osteosarcoma cells).

U2OS were a kind gift from C. Azzalin (Instituto de Medicina Molecular, Portugal, cells originated from A. Londono Vallejo).

#### Primary human fibroblasts.

Human primary foreskin fibroblasts were kindly provided by Dr. Hans-Dietmar Beer, University of Zurich, Switzerland. The foreskin had been collected with informed written consent of the parents in the context of the Biobank project of the Department of Dermatology, University of Zurich, and its use had been approved by the local and cantonal Research Ethics Committees. Cells were used at passage number 6 and 7.

#### Cell-cycle synchronization.

HeLa cells were synchronized using one of two protocols:

Protocol 1 was used in Figures 2A and B, 3B and D, 4B, C, and E, 5. Here, a double thymidine (2 mM, Sigma Aldrich; St. Lewis, Missouri) treatment was done. Cells were treated with thymidine for 16 h, released for 8 h and treated with thymidine a second time for 20 h. 1 h after the second thymidine release, pLacO transfection was performed. 6 h after pLacO transfection cells were washed with 20 U/ml heparin in PBS (3 × 3 min, 37°C).

Protocol 2 was used in Figure 7 and Supplemental Figure 7. Here, cells were treated with thymidine (2 mM, Sigma Aldrich) for 16 h, then released for 8 h. 1 h after this release, plasmids (GFP, GFP-LEM) were lipofected. Cells were exposed to a second thymidine treatment (2 mM) for 18 h. Cells were electroporated with pLacO 2 h after the second thymidine release.

### Plasmid

#### Oligonucleotides used

 

**Table:** 

internal Lab ID	Sequence 5′-3′
OLIGO273	CCCAAGCTTCTGATTCTGTGGATAACCGTATTAC
OLIGO274	TCCCCCGGGTAAGATACATTGATGAGTTTGG
OLIGO320	GGAATTCCCATGACAACCTCCCAAAAG
OLIGO309	GGTGGATCCCTACAAGAAG
OLIGO328	CTAGCTAGCATGGTGAACGTGAAGC
OLIGO329	CGGGGATCCCAGGCTGCTTCTGGACACCT
OLIGO330	CAGCCATGCTGGTGGCCA

 

#### Plasmid preparation.

Plasmid DNA was extracted from *Escherichia coli* bacteria (XL1Blue strain (genotype: recA1 endA1 gyrA96 thi-1 hsdR17 supE44 relA1 lac [F´ proAB lacIq Z∆M15 Tn10 (Tetr)]) or DH5α strain (genotype: F– φ80lacZΔ M15 Δ (lacZYA-argF) U169 recA1 endA1 hsdR17 (rK– mK+) phoA supE44 λ- thi–1 gyrA96 relA1), or Stbl2 strain (genotype: endA1 glnV44 thi-1 recA1 gyrA96 relA1 Δ(lac-proAB) mcrA Δ(mcrBC-hsdRMS-mrr) λ- gal F’[proAB+ lacIq lacZΔM15 Tn10]) using plasmid extraction kits (QIAGEN; Venlo, Netherlands or Macherey Nagel; Düren, Germany). The DNA was purified using either Phenol/Chloroform/Isopropanol, ethanol, or 2-Propanol purification. The purified DNA pellet was resuspended in ddH_2_O of appropriate volume. Plasmid concentration was measured by a NanoDrop Spectrophotometer (Thermo Fisher Scientific).

#### Construction of plasmids.

pControl 1 is also termed pSR9vector-CMV-mCherry (internal Lab ID: PLA1036): CMV-mCherry-SV40-PA was amplified via PCR and the OLIGO273 and OLIGO274 from p-mCherry-N1 without the multiple cloning site (modified, originally from Clontech, Takara; United States. Internal Lab ID: PLA1029). The PCR product for CMV-mCherry was cloned into the backbone of pLacO (internal Lab ID: PLA977), after removing the LacO repeats.

pLacI-mCherry (no NLS; internal Lab ID: PLA1107): LacI was amplified with PCR from LacI-NLS-mEGFP (internal Lab ID: PLA978) using OLIGO328 and OLIGO329 with restriction sites for NheI and BamHI. The backbone vector pIRESpuro2-FLAG-mCherry (internal Lab ID: PLA768, kindly gifted from Yves Barral [IBC, ETH Zurich, Switzerland]) was digested with NheI and BamHI and LacI PCR insert was ligated. Clones were checked with sequencing using OLIGO330.

pEGFP-LEM-nes (internal Lab ID: PLA1098): The sequence of human Emerin’s LEM-domain with a nuclear exclusion signal (nes)(GGAATTCCTCCGAAGATATGGACAACTACGCAGATCTTTCG

GATACCGAGCTGACCACCTTGCTGCGCCGGTACAACATCCCGCACGGGCCTGTAGTAGGATCAACTCGTAGGCTTTACGAGAAGAAGATCTTCGAGTACGAGACCCAGAGGCGGCGGGCCCGGGATTTAGCCTTGAAATTAGCAGGTCTTGATATCTACCCCGAAGATTAAGCGGCCGCTAAACTAT) (internal Lab ID: SYN2) was ordered from Lifetechnologies AG (Basel, Switzerland) and inserted into a modified version of pEGFP-N1 (internal lab ID: PLA328).

pEGFP-BAF (internal Lab ID: PLA1089): BAF was amplified by PCR from pEGFP-HIS-BAF (internal Lab ID: PLA1080; was a kind gift from Tokuko Haraguchi [National Institute of Information and Communications Technology 588-2 Iwaoka, Iwaoka-choNishi-ku, Kobe 651-2492, Japan]) with the primers OLIGO320 and OLIGO309, digested with EcoRI and BamHI, and inserted into pEGFP-HIS-BAF (internal Lab ID: PLA1080).

#### Plasmid transfection

##### Polymer-based transfection.

Plasmid was transfected into cells using X-tremeGENE 9 DNA Transfection Reagent (Roche; Basel, Switzerland). The plasmid:transfection reagent ratio (w:v) was 1:3. Plasmid DNA concentration was either 25 ng (Figures 2, A and B, 3–5, 8 and 9; Supplemental Figures 5, C (HeLa), E and F, 8), 100 ng (Figures 1, 2C, 6, A–D, 7, Supplemental Figures 1–3, 5, 6A and 7), or 330 ng (Supplemental Figure 5 MDCK part) per cm^2^ cell culture dish area. For double transfections (Figures 4, E–G, 6, Supplemental Figure 6C) plasmids were mixed in a 1:1 ration and transfected at total 100 ng/cm^2^ cell culture dish area. To wash away excess transfection mix, cells were washed with 20 U/ml heparin (Sigma Aldrich, Switzerland) 6 h after transfection in Figures 3, B–D, 4, A–D, Supplemental Figure 6B. In all other conditions, the transfection mix was left to incubate with the cells for the time mentioned.

##### Electroporation.

Electroporation was conducted by a MicroPorator (AxonLab; Baden, Switzerland) with Neon-Transfection system 10 µl Kit (invitrogen; Waltham, Massachusetts, United States). The electroporation parameters were 1000 V, 30 ms and 2 pulses for 10 µl electroporation tips using 250 ng DNA per 10^5^ suspension cells in R-buffer. Electroporated cells with same condition were collected in one tube and then seeded on cover slips (Figure 7, Supplemental Figures 1B, 5, A and B, 7). In average, our electroporation conditions introduce less DNA per cell than polymer-based transfection, which is reflected in the size of the plasmid foci generated by these methods (Supplemental Figure 1).

#### Plasmids used

**Table:** 

Plasmid	Source	internal Lab ID	Used in
pEGFP-BAF	this study	PLA1089	Figure 3, C and E
pEGFP-C1	Clontech, Takara	PLA240, PLA997	Figures 4, E–G, 7, Supplemental Figure 7, B–E
pEGFP-HIS-BAF	T. Haraguchi (Shimi *et al.*, 2004)	PLA1080	Cloning of PLA1089
p-EGFP-IBB	D. Gerlich (Schmitz *et al.*, 2010)	PLA1061	Figure 4C
p-EGFP-KDEL	A. Helenius (IBC, ETH Zurich, Switzerland)	PLA936	Figure 2B
pEGFP-LEM-NES	this study	PLA1098	Figure 7, Supplemental Figure 7, B–E
pEGFP-N1	Clontech, Takara, USA	PLA328	Cloning of PLA1098
pEGFP-N3-RCC1	Y. Zheng (Li *et al.*, 2003)	PLA1074	Figure 4D
p-EGFP-POM121	J. Ellenberg (Beaudouin *et al.*, 2002)	PLA1071	Figure 4B
pIRESpuro2-FLAG-mCherry-LacI	this study	PLA1107	Figures 4, E–G, 5
pIRESpuro2-FLAG-LacI-NLS-mCherry	Y. Barral (IBC, ETH Zurich, Switzerland)	PLA976	generation of stable cell line HeLa LacI-NLS-mCherry
pLacI-NLS-mEGFP	(Wang *et al.*, 2016)	PLA978	Figure 6
pLacO	S. M. Gasser, (Rohner *et al.*, 2008), as in (Wang *et al.*, 2016)	PLA977	Figures 1–9, Supplemental Figures 1–8
p-mCherry-Sec61β	T. Kirchhausen; (Lu *et al.*, 2009)	PLA948	Figures 2A, 8C
p-mCherry-N1 without multiple cloning site	modified from Clontech, Takara, USA		Cloning of PLA1036
pMLBAD (pControl2)	A. Nägeli (Lefebre and Valvano, 2002)	PLA1069	Supplemental Figure 5, D–F
pSR9vector-CMV-mCherry (pControl1)	this study	PLA1036	Supplemental Figure 5, D–F

 

### FISH

#### FISH probes.

**FISH probes of PNA quality** (Figure 8, 9, Supplemental Figure 8)

**Table:** 

probe	5′ end fluorescent label	Sequence 5′–3′	Company
TelG	Tamra	TTAGGGTTAGGGTTAGGG	Biosynthesis
TelC	Cy5	CCCTAACCCTAACCCTAA	Panagene
LacO	Alexa 488	GAATTGTGAGCGGATAACAATT	Panagene
scramble	Alexa 488	GGGTAGGAGGTTAGTGTTTTGAGT	Panagene

Other FISH probes were generated with nick-translation method, with Alexa 568-dUTP (Invitrogen), according to manufacturer’s instructions on indicated template DNAs as depicted in Supplemental Figure 5D.

#### DNase I enzyme treatment before FISH.

U2OS and HeLa K cells were fixed with methanol (Supelco; Bellefonte, Pennsylvania, United States) for 10 min at −20°C and then washed three times in onex PBS. Cells were permeabilized with 0.5% Triton X-100 (Sigma-Aldrich, St. Louis, Missouri, United States) for 10 min, then incubated with 0.5 unit/µl DNase (BioConcept; Allschwil, Switzerland) in 1 × PBS for 2 to 2.5 h at 37°C.

#### Regular FISH.

Method modified from (Lansdorp *et al.*, 1996). Cells were rinsed briefly in PBS before fixation. The cells were fixed in 2% paraformaldehyde (Polyscience; Hirschberg an der Bergstrasse, Germany) in onex PBS pH 7.4 for 10 min at room temperature (RT) or in 100% methanol for 10 min at −20°C. Cells were rinsed in onex PBS three times for 5 min and fixed again for 10 min in methanol at −20°C if they were fixed with 2% PFA before. Cells were permeabilized with 0.2% Triton X-100 for 10 or 20 min, then treated with PBS containing 20 mg/ml RNase (Thermo Fisher Scientific) at 37°C for 30 min to 1 h. PNA probes were diluted to 20 nM concentration in hybridization solution (70% deionized formamide [Eurobio; Paris, France], 0.5% blocking reagent [Roche], 10 mM Tris-HCl [pH 7.2]). The DNA was denatured at 80°C for 3 or 15 min. And then incubated in a humid chamber in the dark for 2 h at RT. Cells were washed with hybridization wash solution 1 (10 mM Tris-HCl [pH 7.2], 70% formamide and 0.1% BSA [Gerbu; Gaiberg, Germany]) for two times, 15 min each time at RT and with hybridization wash solution two (100 mM Tris-HCl [pH 7.2], 0.15 M NaCl and 0.08% Tween-20 [Sigma-Aldrich]) for three times. The nuclei were stained by Hoechst33342 (Thermo Fisher Scientific) for 3 min at RT in 1 × PBS and rinsed once with 1x PBS. Cover slips containing cells were mounted in Mowiol 4-88 (Sigma Aldrich) containing 1.4% wt/vol DABCO (Sigma Aldrich), sealed with nail polish. This method was applied for the results shown in Figure 8, A and B, Supplemental Figures 5, E and F, 8, A–E.

#### FISH-IF.

After the RNase treatment, cells were blocked with 5% BSA in 1 × PBST for 1 h at RT. Then cells were incubated in primary antibody diluted in 1% BSA in 1 × PBST in a humidified chamber for 1 h at RT. Incubation with the secondary antibody (1:500 for each) in 1% BSA / 1 × PBST for 1 h at RT in the dark. Cells were fixed with 4% paraformaldehyde for 7 min, and then PFA was quenched with 5% BSA in 1 × PBS and 20 mM glycine for 30 min. Cells were hybridized with probes as described above. This method was applied for the results shown in Figures 8, C and E, and 9, Supplemental Figure 8, F–H.

### Immunofluorescence

#### Antibodies used

 

**Table:** 

Antibodies	Source	host	fixation	dilution	Identifier	internal Lab ID
monoclonal NPC (Mab414)	Abcam	mouse	MeOH	1:1000 (U2OS) or 1:2000 (HeLa)	ab24609	AB324
Lap2β	BD transduction laboratories	mouse	formaldehyde	1:500	611000; 27/LAP2	AB273
polyclonal Emerin	Abcam	rabbit	formaldehyde	1:500	ab40688	AB286, AB321
serum LEM4	Ian Mattaj, (Asencio *et al.*, 2012)	rabbit	formaldehyde	1:1000	BCFED3 20.1.10	AB282
polyclonal LBR	abcam	rabbit	MeOH		ab122919	AB264
serum ELYS/MEL-28	Iain Mattaj (Franz *et al.*, 2007)	rabbit	formaldehyde	1:200	N/A	AB304
IgG, Alexa-Fluor^TM^ 647	ThermoFischer Scientific	mouse	-	1:500	A21236	AB251
IgG, Alexa-Fluor^TM^ 594	ThermoFischer Scientific	mouse	-	1:500	A11032	AB250
IgG, Alexa-Fluor^TM^ 647	ThermoFischer Scientific	rabbit	-	1:500	A21245	AB316
IgG, Alexa-Fluor^TM^ 594	ThermoFischer Scientific	rabbit	-	1:500	A11037	N/A
IgG, Alexa-Fluor^TM^ 488	ThermoFischer Scientific	rabbit	-	1:500	A11034	AB252

 

#### Immunofluorescence staining.

Cells in Figure 3B were fixed 30 h after pLacO transfection, cells in Figure 6 were fixed 48 h after transfection, else cells were fixed 24 h after pLacO transfection. Cells were either fixed with methanol at −20°C for 6 min, or with 1 or 4% paraformaldehyde for 10 min at RT. Cells were permeabilized for 5 or 10 min with 0.2 or 0.1% TritonX-100 at RT. Blocking was performed with 5% BSA (Boehringer Mannheim, now Roche) in onex PBST (onex PBS with 0.05% Tween-20) for 1 h at RT. Cells were then incubated with primary antibodies diluted in blocking buffer for 1 h. Followed by incubation with secondary antibodies diluted in blocking buffer for 45 min to 1 h. Cells were then stained with 2 µM Hoechst33342 (Molecular probes, Thermo Fisher Scientific) for 10 min and mounted in Mowiol with 1.4% wt/vol DABCO. Cover slips were sealed with Nail polish (Lucerna Chem AG; Luzern, Switzerland) and stored at 4°C.

### Image acquisition

Imaging was done at the Scientific Center for Optical and Electron Microscopy (ScopeM, ETH Zurich).

#### Fixed cell imaging.

For images of fixed cells, z-stacks minimally encompassing entire cells were acquired in 0.3 µm or 0.2 µm steps using a 60x NA 1.42 objective on a DeltaVision personalDV multiplexed system (epifluorescence based IX71 (inverse) microscope; Olympus; Tokio, Japan) equipped with a CoolSNAP HQ camera (Roper Scientific; Planegg, Germany).

**Table:** 

Figure	Cell line	Microscope	Objective	Settings
Figure 1, A–D, Supplemental Figures 1, 2, and 3	HeLa LacI control (LacI-NLS-GFP)	Nikon Wide Field microscope (Nikon Ti2-E; Nikon, Tokio, Japan)	S Fluor 20 × NA 0.75 DIC N2 WD 1.0 mm	Z-stacks with 21 slices × 0.3 µm
Figure 6, Supplemental Figure 6F	Primary fibroblasts transiently expressing LacI-NLS-GFP	Nikon Wide Field microscope	Plan Apo lambda 60 × NA 1.4 oil WD 0.13 mm	Z-stacks with 41 slices × 0.3 µm Dapi (Hoechst, DNA) channel was used as reference and the chromatic offset in mCherry and GFP channels was corrected for.
Figure 7, Supplemental Figure 7	HeLa LacI-NLS-mcherry	DeltaVision personalDV Multiplexed (epifluorescence based IX71 (inverse) microscope; Olympus; Tokio, Japan)	60 × 1.42NA DIC Oil PlanApo Objective pco.edge 5.5 camera	z-stacks with 0.3 µm steps

 

#### Live-cell microscopy

 

**Table:** 

Figure	microscope
Figure 1A, Supplemental Figures 1, 2, and 3	Visitron Spinning Disk (Nipkow spinning disk setup with Nikon Eclipse T1 (inverse) microscope, equipped with 2x EMCCD Andor iXon Ultra cameras, LUDL BioPrecision2 stage with Piezo Focus, Carl Zeiss Microscopy; Jena, Germany).Nikon Wide Field (Nikon Ti2-E equipped with a fast xy stage (Prior), a piezo z-drive (Prior) and a NIDAQ board)
Figure 1E	Visitron Spinning Disk
Figures 2, A and B	Visitron Spinning Disk
Figure 3C	Visitron Spinning Disk
Figure 4E	Visitron Spinning Disk
Figure 5	Visitron Spinning Disk
Supplemental Figures 2 and 3	Visitron Spinning Disk (e1, e2) and Nikon Wide Field (e3, e4)
Supplemental Figure 4	Visitron Spinning Disk

For live-cell microscopy, three cell lines were used: Control HeLa, HeLa-LacI, or HeLa K cells stably expressing aa 244– 453 of Lap2β-GFP transiently overexpressing LacI-mcherry.

For results displayed in Figures 1E, 2, A and B, 3C, 4E, 5; Supplemental Figure 4, cells were seeded on Lab-Tek II chambers (Nunc, Thermo Scientific) with CO_2_-independent media (Life Technologies) containing 10% FCS and incubated at 37°C on a Spinning Disk microscope (Nipkow spinning disk setup with Nikon Eclipse T1 (inverse) microscope, equipped with 2x EMCCD Andor iXon Ultra cameras, LUDL BioPrecision2 stage with Piezo Focus, Carl Zeiss Microscopy; Jena, Germany). For imaging in Figure 5 the GFP-Like (Em 520/35) and DsRed-like (Em 617/73) Emission Filter Wheels and 2x Evolve 512 cameras (Photometrics; Tucson, Arizona, United States) were used. For long-term time-lapse imaging, cells were recorded every 15 min (Figure 5) or 30 min (Figure 1E) or 45 min (Supplemental Figure 4) in z-stacks (33 × 0.7 μm steps using a 63 × 1.2 NA objective). To monitor cell contours, cells were illuminated with transmission light with single z-focus. For some still images, cells expressing Sec61-mCherry (Figure 2A), eGFP-KDEL (Figure 2B), and eGFP-BAF (Figure 3C) were imaged after incubation with 2 µM Hoechst33342 for 10 min, using a DeltaVision microscope (DeltaVision personalDV system [epifluorescence based IX71 {inverse} microscope; Olympus]).

For results displayed in Figure 1, Supplemental Figures 2 and 3, HeLa Control cells were seeded on ibidi eight-well chambers (ibidi µ-Slide eight well ibiTreat, Gräfelfing, Germany). 24 h after seeding, cells were incubated at 37°C with 5% CO_2_ (OkoLab, Pozzouli NA, Italy) either at the Visitron Spinning Disk (experiments e1 [internal Lab ID: EXP345] and e2 [internal Lab ID: EXP337]) or a Nikon Wide Field microscope (Nikon Ti2-E [inverse], experiments e3 and e4 [internal Lab ID: EXP604]). For Visitron spinning disk imaging a GFP-Like (Em 520/35) Emission Filter Wheel and 2x Evolve 512 cameras (Photometrics) were used. Bright field imaging was done with the coolLED *p*E-100 control system (coolLED; Andover, Great Britain). For Nikon Wide Field imaging the GFP (Em 515/30) Emission Filter Wheel or bright field presetting was used. For detection, the Orca Fusion BT (Hamamatsu; Shizuoka, Japan; 2304 × 2304 pixels, 6.5 µm × 6.5 µm) system was used. For each experiment at the Visitron Spinning Disk microscope, 5 regions of interest (ROI) were imaged. For each experiment at the Nikon Wide Field microscope, 6 ROI were imaged. In the live-cell analysis included are only ROI with 0.9766 cells/pixel, thus one ROI of e1 at the Visitron Spinning Disk microscope and two ROI of e3 as well as two ROI of e4 at the Nikon Wide Field microscope were excluded. Cells were recorded every 30 min as z-stacks (22 × 0.7 μm steps using 20 × 0.75 CFI Plan Apo VC at the Visitron Spinning disk and 22 × 0.7 μm steps using S Fluor 20 × NA 0.75 DIC N2 WD 1.0 mm at the Nikon Wide Field). On both microscopes, cells were lipofected with pLacO using X-tremeGENE 9 DNA Transfection Reagent (Roche). The plasmid:transfection reagent ratio (w:v) was 1:3 with a plasmid DNA concentration of 100 ng/cm^2^. The lipofection mix remained on the cells during imaging. The death rate was low at both microscopes (Supplemental Figure 2A).

#### FRAP.

FRAP experiments (Figure 4, E–G) were performed using a modified method that was previously reported (Clay *et al.*, 2014). 24 h after pLacO transfection, live HeLa-LacI cells (seeded on a Lab-TekTM II chamber, CO_2_-independent media, 37°C incubator) and free eGFP were imaged on a confocal microscope (LSM 760; Carl Zeiss Microscopy) with a Plan Apochromat 63× /1.4 NA oil immersion objective. The ZEN software (Carl Zeiss Microscopy) was used to control the microscope. eGFP emission was detected with a 505 nm long pass filter. Photobleaching was applied on a region of interest (cluster and then control area) as indicated in Figure 4, E–G. Bleaching was applied with 50–100 iterations using 30–50% laser power, but always with the same settings between the cluster and control area in each cell.

#### Correlative light and electron microscopy (CLEM).

HeLa K cells stably expressing LacI-NLS-mCherry were cultured on a 3.5 cm glass bottom dish with grid (MatTek; Ashland, Massachusetts, United States) and transfected with pLacO for 24 h. Cells were processed as described in (Wang *et al.*, 2016).

### Data Analysis (Fiji, Prism, Diatrack, etc.)

#### Image processing.

Images acquired from DeltaVision (Olympus) microscope (Figures 2C, 3, B–D, 4, A–D, 8, Supplemental Figures 5, A and E, 6A, 7, C and D, 8, A, B, and D, 9A) were deconvolved using Softworx (Applied Precision; Rača, Slowakia). Images acquired from LSM 710 confocal microscope were deconvolved using Huygens Software (Scientific Volume Imaging; Hilversum, Netherlands) before correlating with EM images (Figure 2A). The correlation analysis between confocal and EM images (Figure 3A) were performed using Amira software (FEI/Thermo Fisher Scientific), as in (Wang *et al.*, 2016). General, the presented images are single z-slices or if indicated projections of multiple z-slices images.

Images in Figure 3A (confocal image), 6 and Supplemental Figure 6F were deconvolved using Huygens (Scientific Volume Imaging).

#### Image analyses.

Images were analyzed using Fiji 1.51n Software.

##### Colocalization.

For colocalization analyses, the overlay of the reporter fluorescence and LacI fluorescence was used (Figures 2C, 6, 8; Supplemental Figures 6, D and E, 8). The qualitative classes for reporter molecules “enriched”, “nonenriched”, “present”, and “absent” are established applying the following rules:

For experiments presented in Figure 3 and Figure 4:

Generally, marker fluorescence intensities were used to qualitatively determine colocalization of markers with the plasmid focus with the following criteria.

“Nonenriched”: plasmid foci with marker fluorescence signal in the z-stack slice in, directly underneath or above the position of the plasmid focus, and/or marker fluorescence signal in xy-direction adjacent. The intensity of the marker fluorescence is like the cytoplasmic-marker fluorescence (relative readout to the intensity of the rest of the cell).

Plasmid foci with marker “enriched” have marker fluorescence at same positions described in “nonenriched”, but with higher intensities compared with the reference marker fluorescence of the respective marker (i.e., Emerin at NE or in ER; LEM4 in ER). Where sensible, results state the two “enriched” reference marker fluorescence (i.e., ER or NE).

Category “present” (Figures 4 and 8) encompasses “enriched” and “nonenriched.”

Plasmid foci with marker “absent” neither have marker fluorescence in the adjacent slides, underneath or above the position of the focus, nor a marker-fluorescence signal in xy direction adjacent to the focus nor in the sliced with the focus.

For data presented in Figure 3E:

For Emerin and Lap2β, the quantitative enrichment factor analysis (below) were back translated into qualitative classification: "enriched” with an enrichment factor > 1, or “nonenriched” with enrichment factor < 1. For BAF and LBR, the classification of “enriched,” “nonenriched,” and “absent” as described above for Figure 3 was used.

For data presented in Figure 6, Supplemental Figure 6C:

In the single z-slice, where the plasmid focus was in focus, a line scan across the biggest axis of the plasmid focus and either the ER (for LEM4) or across the nucleus (for Emerin) was made, displaying the intensity distribution along that line (Fiji, line scan). Classification was according to the following intensity criteria: enriched > NE or > ER: the average intensity of the reporter (Emerin or LEM4) at the plasmid focus was higher compared with the average reporter intensity at the NE or surrounding ER, displayed along the line. Like ER: fluorescence of the reporter (Emerin or LEM4) was in average identical the intensity in the ER surrounding the plasmid focus. Absent: no intensity of the reporter (Emerin or LEM4) at the plasmid focus.

For the experiments presented in Figure 7:

Intensities of reporter proteins (LEM4 or Emerin) at the plasmid focus were visually compared with intensities reporter proteins in the surrounding cytoplasm. “Present”: If the reporter intensity was equal or higher at the plasmid focus than that of the surrounding cytoplasm in the focal slice, directly underneath or above the focus-position of the plasmid focus (0.3 µm distances). Otherwise, the classification was “absent.”

For the experiments presented in Supplemental Figure 7, B and C:

The intensity of Emerin immunofluorescence was measured as RawIntDen (Fiji) in a square (20 × 20 pixel) covering ER and in a same-sized square covering the nucleus of a single cells in maximum intensity projected images. Chosen were in both cases regions where the intensity appeared the most intense as judge by eye. The ratio between the RawIntDen value at the NE divided by that at the ER was calculated for each cell with minimally one cytoplasmic plasmid focus. A ratio above one report about a higher intensity of Emerin (and therefore more Emerin) at the NE compared with the ER of that same cell. A ratio below one represents a higher intensity of Emerin (and therefore more Emerin) at the ER compared with the NE of the same cell.

Quantitative enrichment factor analysis is described in Supplemental Figure 6B.

##### Live-cell imaging analyses

**Plasmid focus localization** (Figure 1, Supplemental Figures 2 and 3):

For plasmid focus analyses, two interphase localizations are classified: cytoplasmic and nuclear. Cytoplasmic plasmid foci: These intracellular LacI-positive plasmid foci are outside of the volume marked by LacI-NLS-GFP fluorescence reporting about the nucleus or are at the cytoplasmic side of the nuclear envelope, which is reported by the outer boarder of the nuclear LacI-NLS-GFP fluorescence, but with minimally 1 pixel of background intensity between the LacI intensity at the plasmid focus and that of the nucleus. Plasmid foci in the nucleus are either nucleoplasmic LacI-positive foci or LacI-positive foci at the nucleoplasmic side of the nuclear envelope, which is reported by the outer boarder of nuclear LacI-NLS-GFP fluorescence. Nucleoplasmic-plasmid foci (nuclear foci) are defined as plasmid foci inside the volume of nuclear LacI-NLS-GFP fluorescence but with an intensity higher than that of the general nuclear LacI-NLS-GFP fluorescence. In addition, the z-slice in which the plasmid focus is most in focus (focal-z-slice) and at highest intensity is also the z-slice, in which nuclear LacI-NLS-GFP fluorescence covers the biggest area. Further, the focal-z-slice of the plasmid lies in between other z-slices in which the general nuclear LacI-NLS-GFP fluorescence is still in focus. Typically, depending on the z-stack spacing, the general nuclear LacI-NLS-GFP fluorescence is still in focus within +/- 1 µm of the focal plane of the plasmid focus. This is to be compared with a situation, where a plasmid focus is at the nuclear envelope and thus in an upper z-slice. In this case the plasmid-focal plane is not identical with the z-slice of the biggest area of general nuclear fluorescence. These classification criteria were used in Figure 1, Supplemental Figures 2 and 3 as well as for classification of plasmid foci being formed in the nucleoplasm in Supplemental Figure 5, G and H. We chose these strict conditions to exclude the option of a false-positive nuclear assignment to plasmid foci.

**Origin-destination analyses** (Figure 1D, Supplemental Figure 2H):

To avoid analyzing plasmid foci that formed in the cytoplasm but in close proximity to the NE, we excluded plasmid foci that formed at the inner side of the nuclear periphery from the analysis focusing thus on foci formed in the inner nucleoplasm. To allow for sorting time the last 25% of the forming plasmid foci in the pooled data set were excluded from this analysis. For the “origin-destination” analysis the location of formation of each plasmid focus was noted (“origin”; either interphase: cytoplasm or nucleoplasm, or during mitosis). Then, after tracing the focus over time until the end of imaging, the location of each plasmid focus at imaging end (“destination”) was noted. If during imaging a cell fused with another cell, died, produced a micronucleus, the nucleus fragmented, the plasmid focus disappeared, or the cell was in mitosis at the end of imaging the focus localization in the last frame before one of these events was noted as the corresponding destination of the given plasmid focus. Only cells that completed a mitosis (first frame with two distinct nuclei in LacI-NLS-GFP channel and two fully divided cells in brightfield channel) were analyzed.

**FRAP quantification** (Figure 4, E and F):

Using Fiji, the mean fluorescence recovery signal was quantified in the bleached area. The fluorescence signal was normalized to that at the beginning of the experiment. All experiments were transferred to Prism software (GraphPad) and fit on an exponential FRAP curve, the mobile fraction was measured by determining the half time (t_1/2_) of fluorescence recovery to reach a plateau level.

**FISH colocalization analysis** (Figures 8 and 9, Supplemental Figure 8):

Fluorescence signals of stained proteins were boosted until the background level of the cell’s cytoplasm was visible. Proteins were noted as colocalizing with tDNA if the protein signals were visually greater than the background in close vicinity to the tDNA focus.

### Statistics

Statistics were conducted using Prism 9.2 (GraphPad) built-in analysis tools.

Figures 3E, 4E, 7, B–D, 8, B and E: Data was tested for normality (Shapiro-Wilk normality test, [α = 0.05]) and analyzed with Welch’s t-tests.

Figures 1C, 9C, Supplemental Figures 5, B, C, and F, 6D, 7C, 8C: Data was tested for normality (Shapiro-Wilk normality test, [α = 0.05]) and analyzed with a paired *t* tests.

Figure 1, B and F: Data was tested for normality (Shapiro-Wilk normality test, [α = 0.05]) and analyzed with a Mann-Whitney test.

Figure 4E (RCC1), Figure 6: data of less than three experiments, no statistical analysis was done.

Supplemental Figure 2, A, G and H: single experiments with only one value, thus no variance and therefore no test is applicable.

Supplemental Figure 2F: no variability, thus no test is applicable.

Supplemental Figure 3B: Data was tested for normality (Shapiro-Wilk normality test, [α = 0.05]) and analyzed with a Wilcoxon matched-pairs signed rank test.

Supplemental Figure 3C: Data was tested for normality (Shapiro-Wilk [α = 0.05]) and analyzed with a Wilcoxon test.

Supplemental Figure 6C: Data was tested for normality (D’Agostino & Pearson normality test, [α = 0.05]) and analyzed with a *t* test.

## Abbreviations used:

BAF: Barrier-to-autointegration factor
ER: endoplasmic reticulum
LEM: LAP2, Emerin, MAN1
NLS: nuclear localization sequence
NPC: nuclear pore complex
