# Supplementary material for: A dedicated cytoplasmic container collects extrachromosomal DNA away from the mammalian nucleus
Source: Mol Biol Cell. 2023 Sep 21;34(11):ar105. doi: 10.1091/mbc.E23-04-0118 (PMC10559310; doi:10.1091/mbc.E23-04-0118)
Supplement: Supplementary file 1 [file mbc-34-ar105-s001.pdf]

Supplementary Materials

Molecular Biology of the Cell

Schenkel *et al*.

Supplemental Figure 1

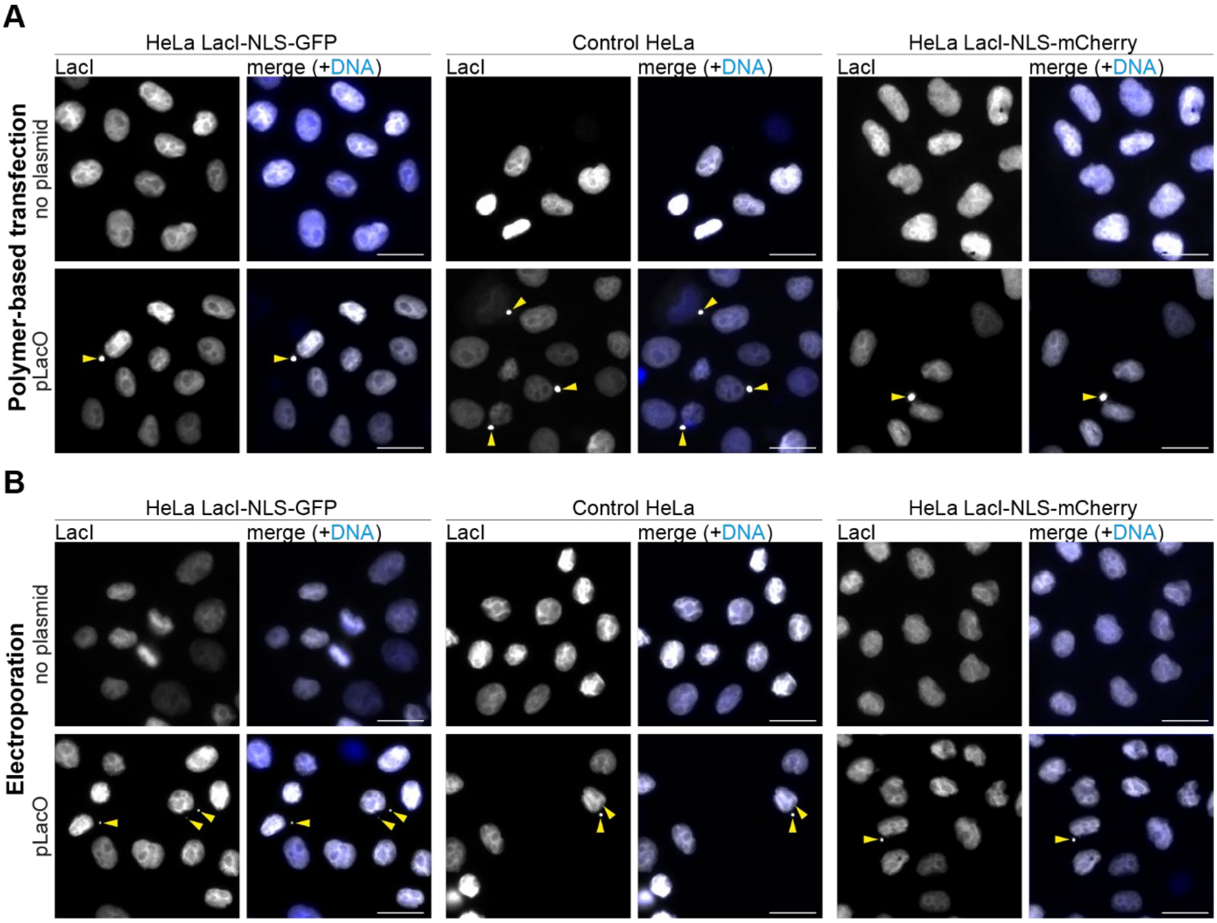

Supplemental Figure 1. Only plasmid-transfected cells have cytoplasmic LacI foci. (A, B) Images of HeLa-LacI cells stably expressing LacI-NLS-GFP or LacI-NLS-mCherry polymer-based transfected (A) or electroporated (B) without or with pLacO and fixed 24 hours after transfection. Scale bar, 20 µm. DNA, blue (Hoechst stain). Plasmid foci in the cytoplasm, yellow arrowheads.

Supplemental Figure 2

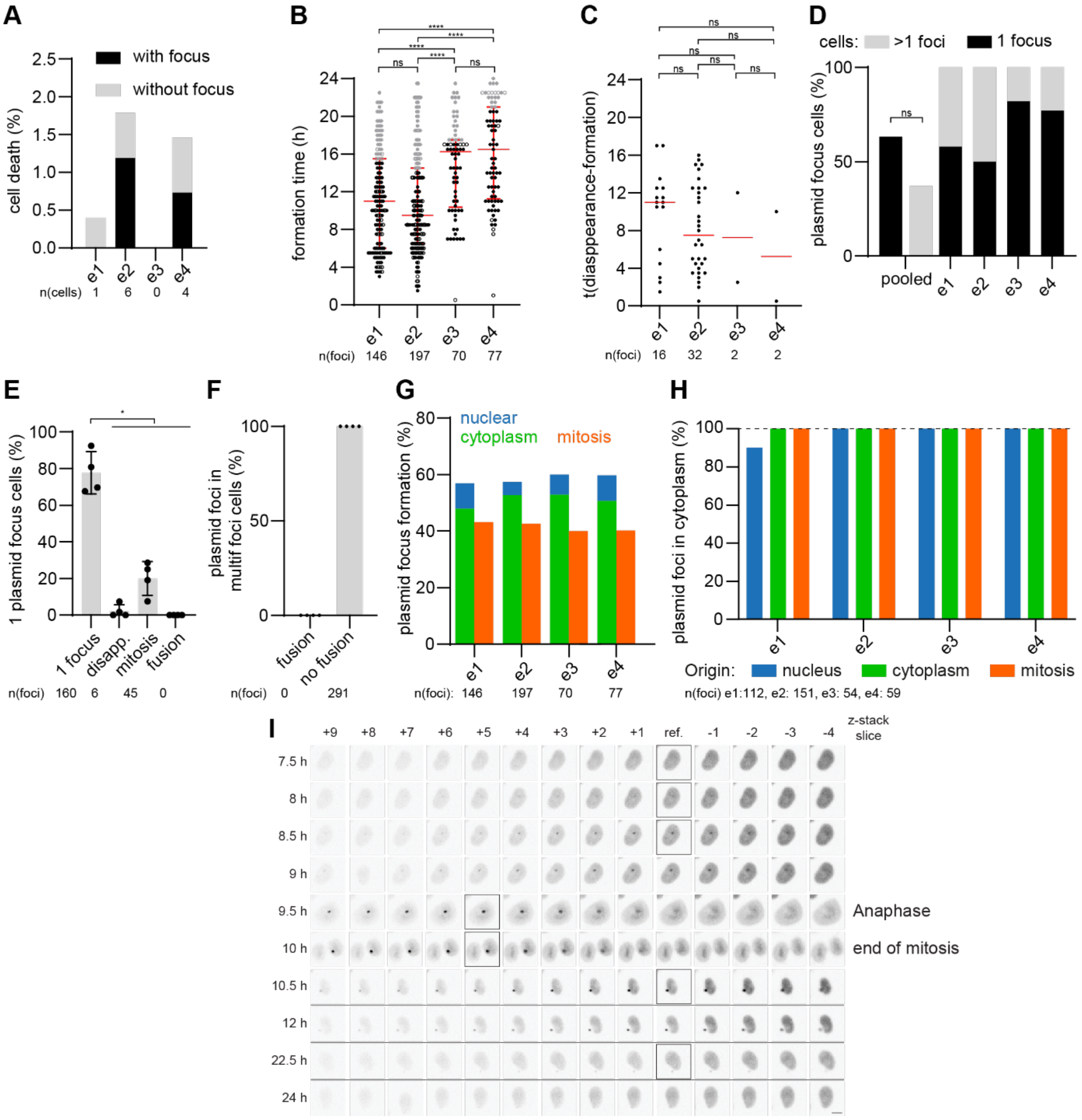

Supplemental Figure 2. Dynamics of plasmid foci in individual live cell imaging experiments. Individual exp., e1 - e4. (A) Cell death events until end of imaging. Cells died without focus, grey; died with focus, black; % relative to cells at end. n(cells): 253, 336, 270, 273. (B) Timing of focus formation. 1 focus, circle; time, after polymer-based transfection; median & interquartile range, red; last 25 % formations, grey. This normal data was tested with Kruskal-Wallis (Dunn's multiple comparisons) test, ns = not significant, **** = p-value <0.0001. (C) Presence period of disappearing foci. 1 focus, circle; median, red line. This normal data was tested with Kruskal-Wallis (Dunn's multiple comparisons) test, ns = ns = not significant. (D) Cumulative frequency of 1-focus cells (black) and multi-foci cells (grey) during imaging period. Maximal number of foci per cell during lifetime of single cells; pooled data, pooled. This normal data was tested Paired t-test, 1 focus vs. >1 focus, ns = not significant, p-value = 0.115 (two-tailed). (E) Analysis if origin of 1-focus cells at imaging end. Cell formed 1 focus or divided propagating it, 1 focus; cell formed multiple foci and all but one disappeared, disapp.; partitioning in mitosis resulted in 1-focus cell(s), mitosis; foci fused, fusion. One exp., circle. 4 exp. n(cells, 1 focus): 46/39/38/37=160, n(cells, disapp): 5/1/0/0=6, n(cells, mitosis): 17/16/9/3=45, n(cells, fusion): 0/0/0/0=0. This normal data was tested Paired t-test: 1focus vs.

all other categories, p-value = 0.017 (two-tailed). (F) Fusion of foci in multi-foci cells. Pooled data. (G) Origin of forming foci. Normalized to all foci formed per exp. (H) Cytoplasmic foci depending on origin. Last 25 % formations excluded. n(foci): 112; 100 % reference, dashed line. (I) Focus formation in the nucleoplasm. Cell as Fig. 1A (nucleoplasmic formation, corresponding images with black squares) with z-slices above and below reference slice (ref.) Scale bar, 10 μm. Time, after polymer-based transfection. Black horizontal lines, skipped time points.

Supplemental Figure 3

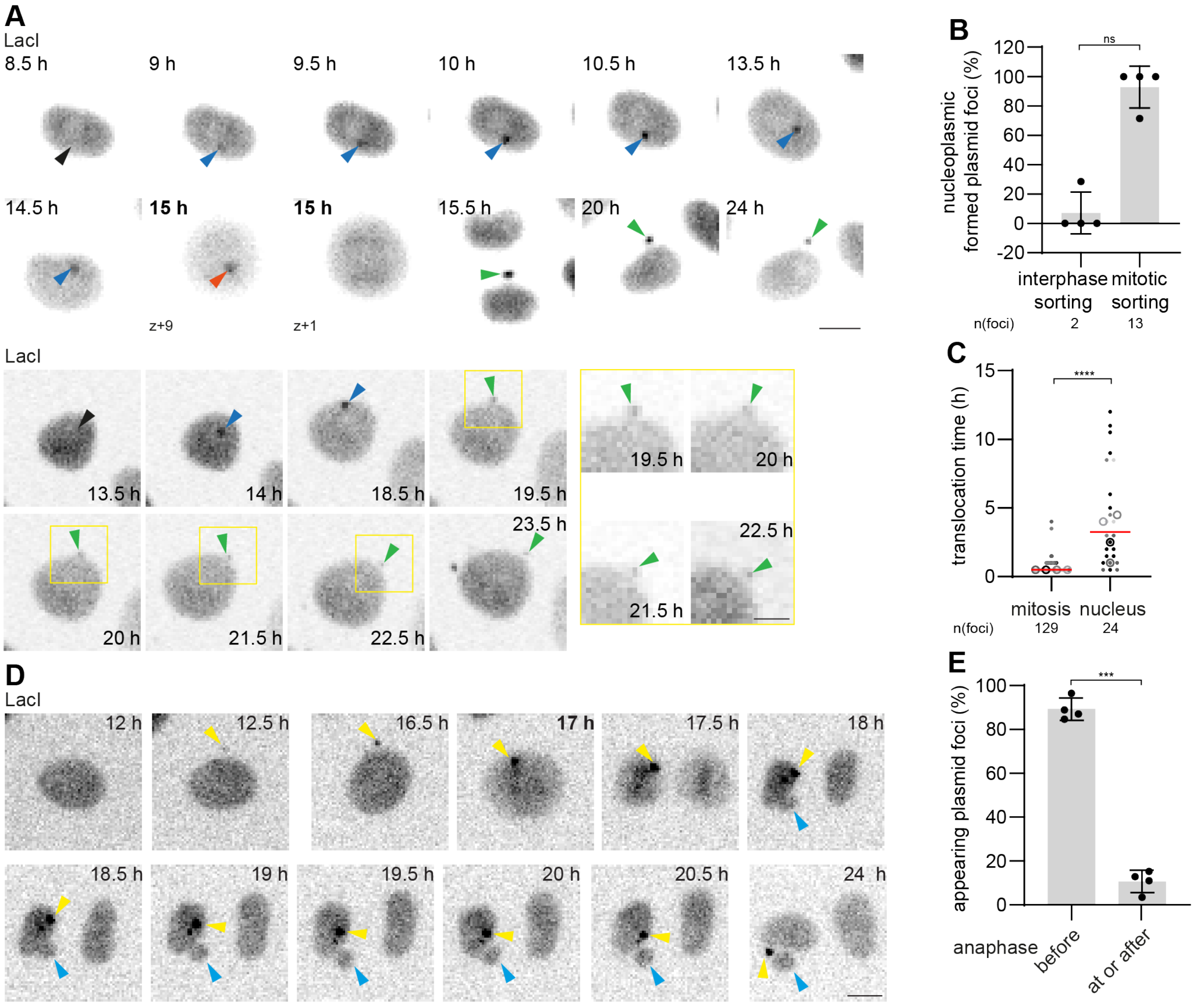

Supplemental Figure 3. How plasmid DNA leaves the nucleus. (A) Two example time-lapse images of focus formations in HeLa-LacI cells; mitotic sorting (upper) and interphase sorting (lower). Scale bar, 10 µm. Time, after polymer-based transfection; bold time, mitosis. Arrowheads: nucleoplasmic, blue; cytoplasmic, green; mitosis, orange; future focus formation area, black; single z-slices. (B) Quantification of events shown in (A). 4 exp., one exp., circle. Mean and SD; only foci analyzed, which appeared in the nucleoplasm but not at the border of the nuclear LacI-NLS-GFP fluorescence neither on nucleoplasmic side (see method); n(foci): 15. This non-normal data was tested with Wilcoxon matched-pairs signed rank test. ns = non-significant. (C) Duration between the first detection of a focus and its first localization in the cytoplasm. Only plasmid foci formed either during mitosis or in the nucleus, appearing during the first 75 % of the formation time of all plasmid foci formations and translocating into the cytoplasm, were analyzed. 1 plasmid focus, filled small circle; 4 exp., exp1, black; exp2, dark grey; exp3, medium grey; exp4, light grey; median, red line; mean of 1 exp, hollow big circle. Color coding same as for plasmid foci. This non-normal data was tested with Wilcoxon test. **** = p-value <0.0001. (D) Time-lapse images contrasting focus (yellow arrowhead) and mitotic micronucleus formations (light blue arrowhead) in HeLa-LacI cells. Scale bar, 10 µm. Time, after polymer-based transfection. (E) Plasmid foci formed during mitosis relative to anaphase. 4 exp. (circles); mean & SD; n(foci): 207. This normal data was tested with a paired t test. *** = p-value 0.0006.

Supplemental Figure 4

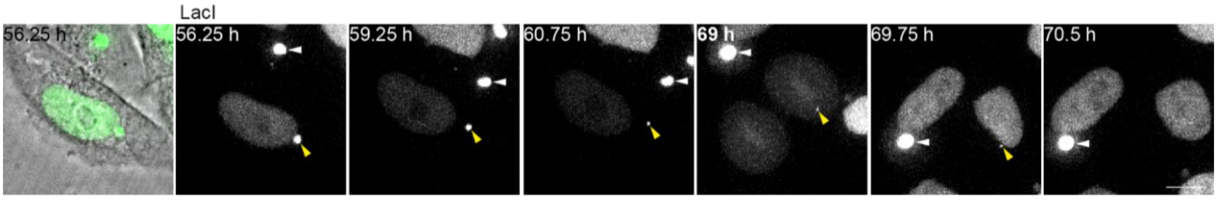

Supplemental Figure 4. Fluorescence dynamics of plasmid foci in long-term movies. Representative time-lapse images of HeLa-LacI cells showing the disappearance of a focus between 56.25 hours and 70.5 hours after transfection. Plasmid focus, yellow arrowheads. Persistent brightness of a focus (white arrowhead) in a neighboring cell shows that the disappearance of fluorescence at a focus is not because of bleaching. Cell outline is shown by transmission light in the first frame (56.25 hours). Scale bar, 10 μm. Time, after polymer-based transfection of pLacO; bold time, mitosis.

Supplemental Figure 5

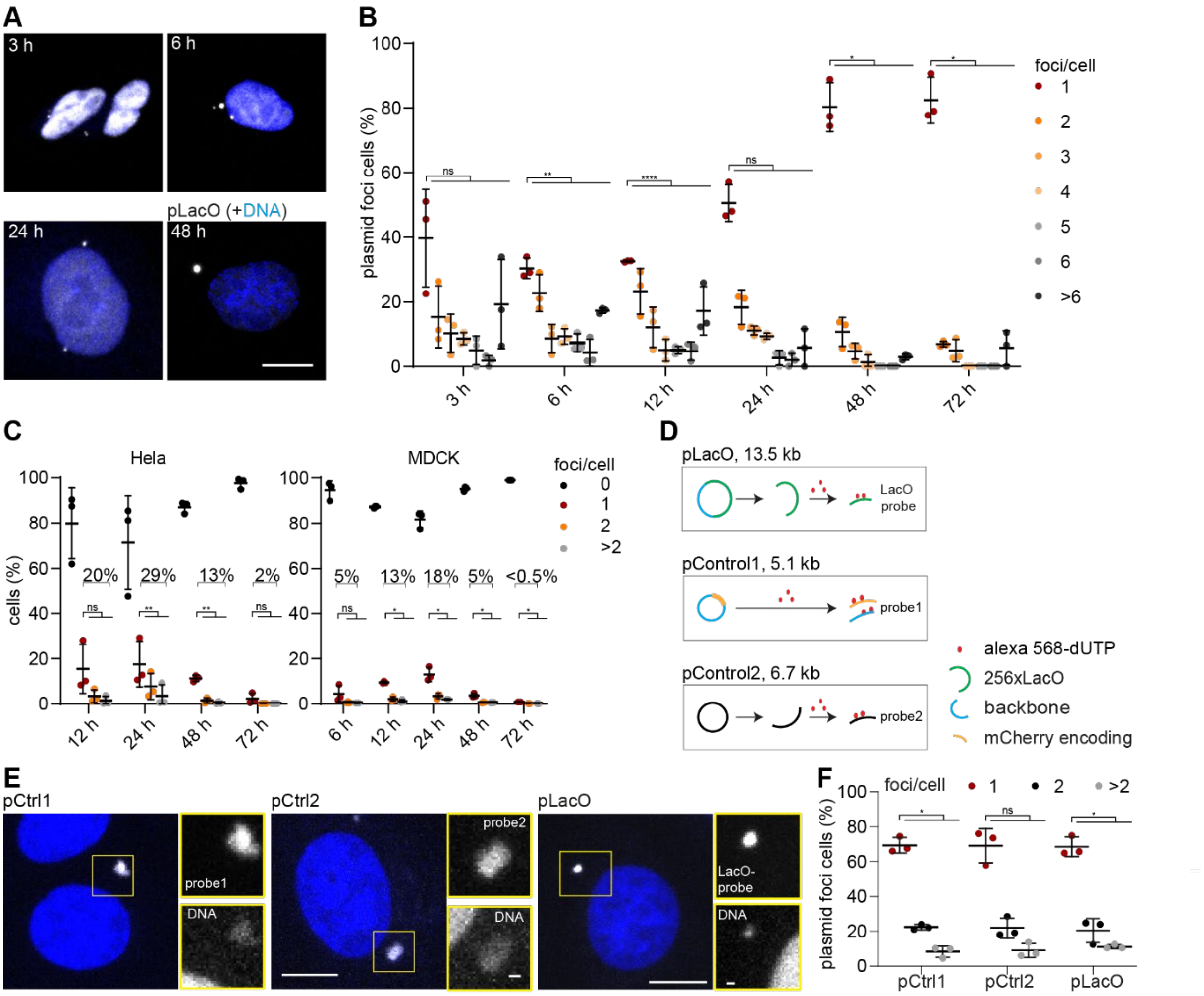

Supplemental Figure 5. Time, transfection method, and plasmid type dependent reaction towards transfected plasmid DNA. (A, B) MDCK-LacI cells electroporated with pLacO fixed, imaged, and analyzed at indicated times after electroporation. (A) Example images. Scale bar, 10 µm. (B) 7 classes (1 - >6) of plasmid foci per cell. 3 exp. 1 exp, circle. Mean & SD; n(cells, 3 hours): 166, n(cells, 6 hours): 162, n(cells, 12 hours): 175, n(cells, 24 hours): 161, n(cells, 48 hours):167, n(cells, 72 hours): 115. This normal data was tested with a paired t-test. ns = non-significant. * = p-value 48 h: 0.0204, 72 h: 0.0156. ** = p-value 0.0086, *** = p-value < 0.0001. (C) 4 classes (0 - >2) of plasmid foci per HeLa-LacI (left panel) and MDCK-LacI (right panel) cell at indicated times after polymer-based transfection. 3 exp. 1exp., circle; Mean & SD. HeLa-LacI: n(cells, 12 hours): 1092; n(cells, 24 hours): 778; n(cells, 48 hours): 1442; n(cells, 72 hours): 7164. This normal data was tested with a paired t-test. ns = non-significant. ** = p-value 12 h: 0.0021, 24 h: 0.0093. MDCK-LacI: n(cells, 6 hours): 3134; n(cells, 12 hours): 1367; n(cells, 24 hours): 1099; n(cells, 48 hours): 4176; n(cells, 72 hours): 17476. This normal data was tested with a paired t-test. ns = non-significant. * = p-value 12 h: 0.0132, 24 h: 0.0438, 48 h: 0.0466, 72 h: 0.0138. (D) Scheme illustrating three transfected plasmids and corresponding FISH probes used in (E, F). (E) Representative images of FISH on HeLa-LacI cells polymer-based transfected with either pLacO (LacO probe), pControl1 (pCtrl1, probe1), or pControl2 (pCtrl2, probe2) 24 hours after transfection. Images are max. intensity-projected z-stacks. Insets: plasmid foci; scale bars: big images: 10 µm; insets, 1 µm. (F) 3 classes (1 - >2) of plasmid foci per HeLa-LacI cell depending on the transfected plasmid. 3 exp, n>50 per exp. This normal data was tested with a paired t-test. ns = non-significant. * = p-value pCrtl1: 0.0173, pLacO: 0.0293.

Supplemental Figure 6

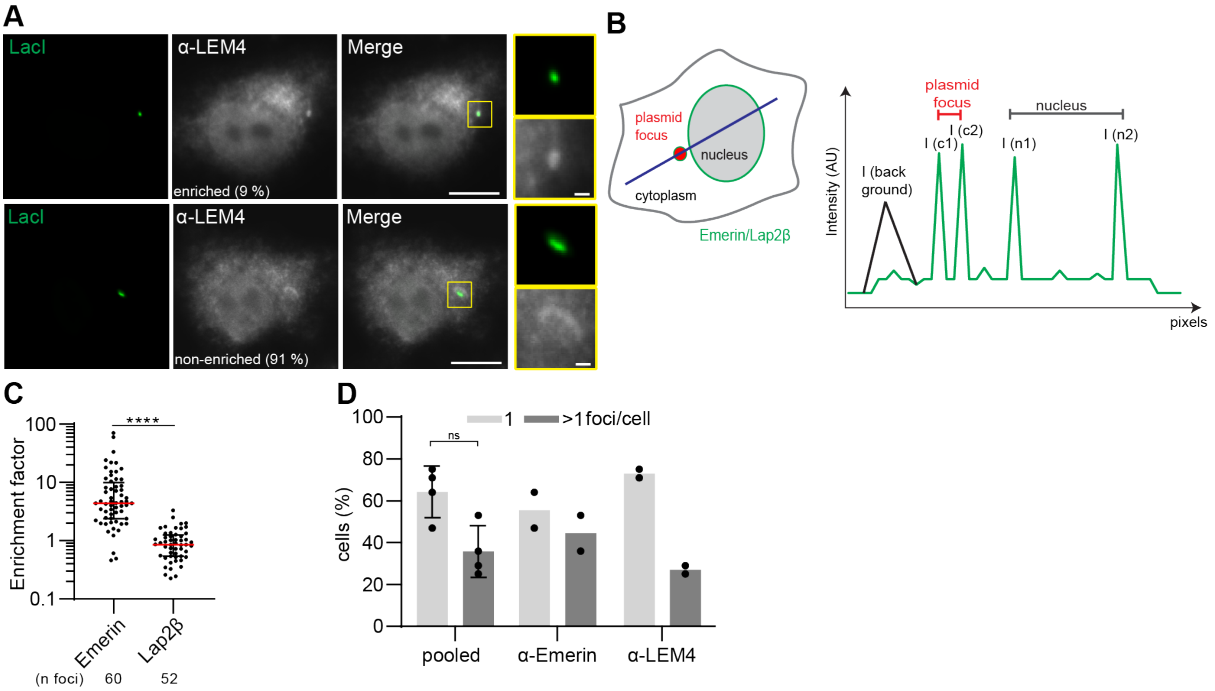

Supplemental Figure 6. Detailed characterization of the exclusome. (A) HeLa-LacI cells electroporated with pLacO and 24 hours later immunostained for LEM4. Single z-slice images; insets: plasmid foci; scale bars: big images,10 µm; insets, 1 µm. Pooled data of 2 exp.; n(cells): 124. (B) : Enrichment factor analysis. Left part: A schematic cell harboring a focus (red) and stained with the reporter of interest (ROI, Emerin or Lap2β, green). Images were analyzed using sing-z slices. The fluorescence intensity of the ROI was measured along a line (blue) crossing the focus and the nucleus. Right part: line-intensity. Along this line, the fluorescent intensities of two brightest pixels at the edges of the focus (I (c1), I (c2)) or the nucleus (I (n1), I (n2)), were averaged. Another averaged intensity of 30-50 pixels along this line, in a cytoplasmic region, was used as background intensity (I (background)). The enrichment factor was calculated as: (Enrichment factor = ((I (c1)+I (c2))*0.5 - I (background))/ ((I (n1)+I (n2))*0.5 - I (background)). (C) A ratio-based fluorescence enrichment analysis for Emerin and Lap2β at plasmid foci in HeLa-LacI cells, 24 hours after pLacO transfection. 3 exp. pooled; plasmid focus, circle. Non-paired t-test with log (value); ****: p<0.0001. (D) Primary human fibroblasts 48 hours after co-polymer-based transfection with pLacO and plasmid encoding LacI-NLS-GFP. Frequency of 1-focus cells and multi-focus cells. 4 exp. 1 exp, circle; merged data from 4 exp, pooled. Single exp. per immunofluorescence stain, α-Emerin or α-LEM4. n(cells, pooled): 106; mean & SD for pooled data. Mean, bar of single stain experiments. This normal data was tested with a paired t-test. ns = non-significant.

Supplemental Figure 7

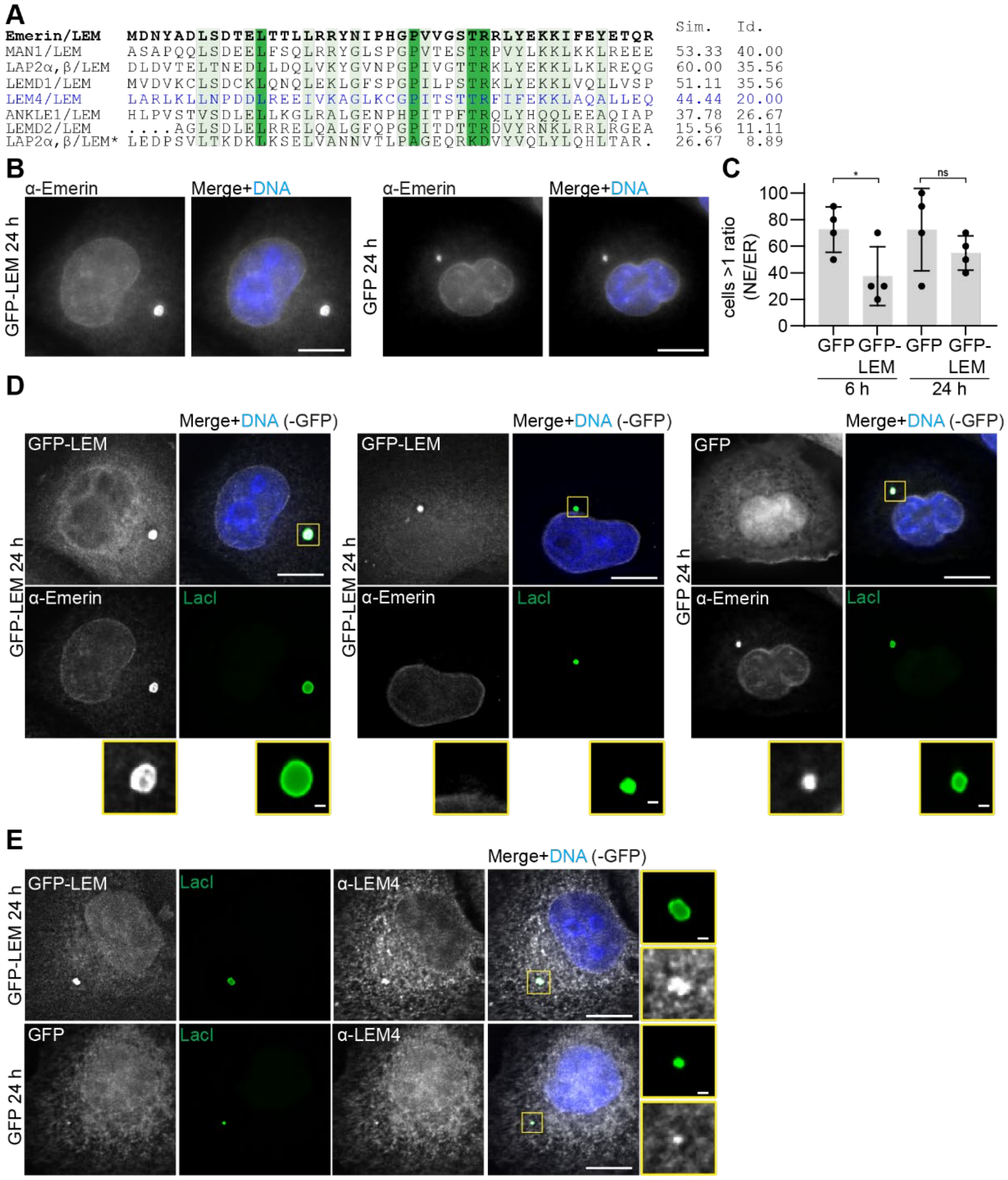

Supplemental Figure 7. Effects of overexpression of Emerin's LEM domain. (A) Amino-acid alignment for the LEM-domain (LEM) and LEM-like-domain (LEM*) of various human LEM-domain proteins. Percentage of similar (Sim.) and identical (Id.) residues compared to Emerin (bold). LEM-domain of LEM4, blue. Sim. residues, light green; Id. residues, dark green. (B-E) HeLa-LacI cells transiently expressing GFP-LEM or GFP 6 hours (C) and 24 hours (B-E) after electroporation with pLacO. DNA, blue (Hoechst staining). (B) Boosted single z-slice images to visualize ER. Same cells as in (D) (left & right panel) immunostained for Emerin. Scale bar, 10 µm. (C) Cells, with a higher measured intensity of Emerin at the NE compared to the ER. 4 exp. 1 exp, circle; mean & SD. This normal data was tested with a Welch's t-test. ns = non-significant, *= p-value 0.3555. (D, E) Deconvolved single z-slice images to visualize plasmid foci. Cells were immunostained for Emerin (D) or LEM4 (E). Insets: plasmid focus. Scale bar: in big images, 10 µm; insets, 1 µm.

Supplemental Figure 8

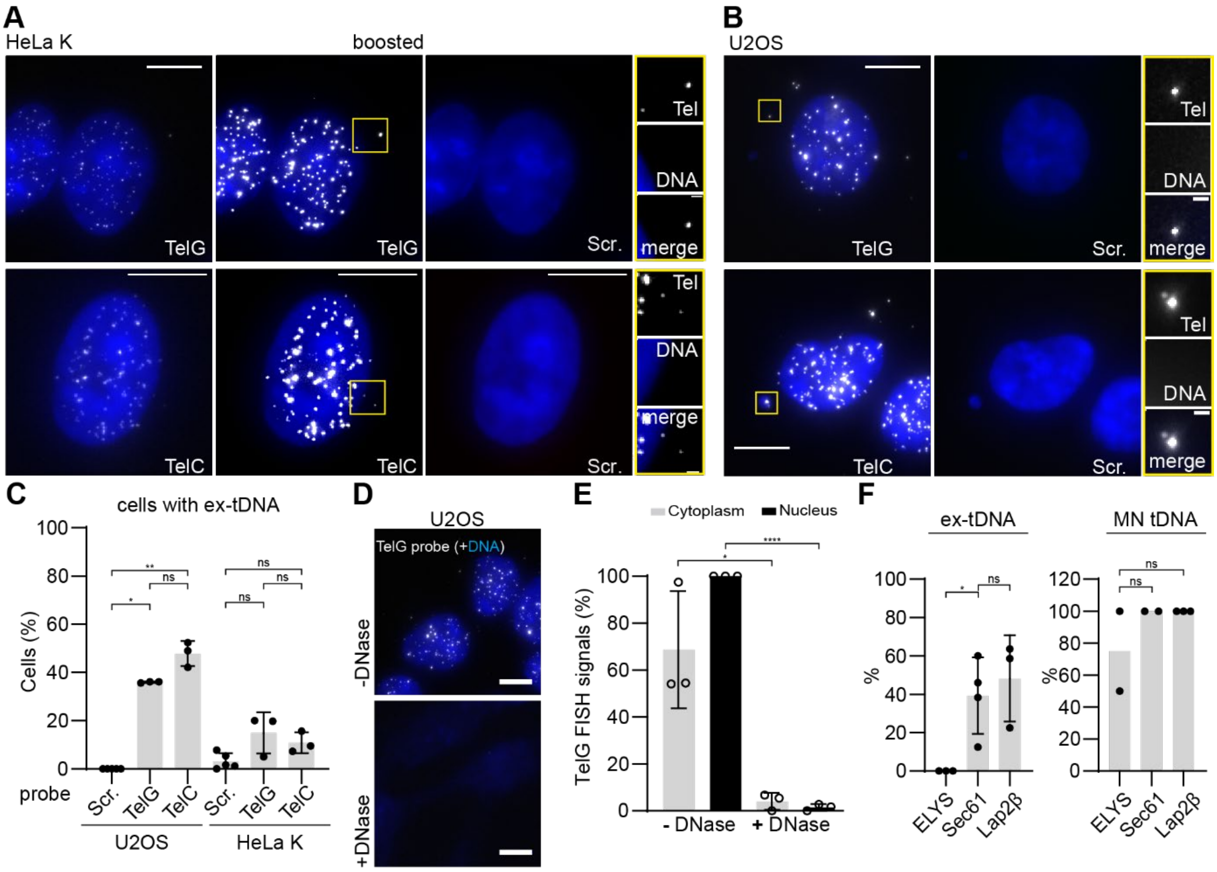

Supplemental Figure 8. Interphase U2OS and HeLa K cells with ex-tDNA. (A, B) Representative max. projected images of HeLa K (A), U2OS (B) cells with TelG or TelC and scramble probes (scr.). Insets: ex-tDNA foci. Imaging conditions in (A, B) were same; corresponding display; except for boosted FISH signals (boosted). Scale bar, 10 µm. (C) Percentages of HeLa K, U2OS cells containing ex-tDNA. One exp (circle), mean & SD. U2OS-TelG: 3 exp; n(cells): 47/73/97=217; U2OS-TelC: 3 exp; n(cells): 57/49/65=171; U2OS-scr.: 5 exp; n(cells): 57/49/65/73/47=291. HeLa-TelG: 3 exp; n(cells): 55/59/96=210; HeLa-TelC: 3 exp; n(cells): 82/64/63=209; HeLa-scr.: 5 exp; n(cells): 82/64/63/55/59=323. This data was analyzed using Kruskal-Wallis test. ns = non-significant. *=p-value 0.0302, **=p-value 0.0029. (D, E) Representative max. projected images (D) and quantification of U2OS (E) with nuclear and cytoplasmic TelG FISH signals, +/- DNase I treatment. DNA, blue (Hoechst stain). Scale bar, 10 µm; mean & SD; 3 exp. (circles). n(-DNase, cytoplasm): 18/20/39=77, n(-DNase, nucleus): 33/37/40=110, n(+DNase, cytoplasm): 2/0/4=6, n(+DNase, nucleus): 1/0/1=2. This data was analyzed using Welch's t-test. *=p-value 0.0437. ****=p-value<0.0001.(F) Colocalization of indicated proteins with ex-tDNA (left) and MN tDNA (right). 3-4 exp. (circles); mean & SD; foci: n(Lap2β, ex-tDNA): 46/77/31=154; n(Lap2β, MN tDNA): 3/7/2=12; n(Sec61, ex-tDNA): 39/13/8/35=95; n(Sec61, MN tDNA): 3/0/1/3=6; n(ELYS, ex-tDNA): 23/27/16=66; n(ELYS, MN tDNA): 3/3/0=6. This normal data was analyzed using Welch's t-test. ns = non-significant. * = p-value 0.0292.
